# New Treatment Strategies for Rare Genomic Alterations: *EGFR* Exon 20 Insertions and *HER2*-Deregulated Lung Cancer

**DOI:** 10.3390/cancers18142334

**Published:** 2026-07-20

**Authors:** Lodovica Zullo, Jordi Remon

**Affiliations:** Gustave Roussy, Department of Cancer Medicine, University of Paris Saclay, 94805 Villejuif, France

**Keywords:** EGFR exon 20 insertions, HER2-mutant, zongertinib, sevabertinib, trastuzumab-deruxtecan, non-small cell lung cancer

## Abstract

Non-small cell lung cancer (NSCLC) comprises a diverse group of malignancies driven by distinct molecular alterations that have become critical targets for precision oncology. Among these, *EGFR* exon 20 insertion mutations and *HER2* dysregulation, including *HER2* mutations, amplification, and overexpression, define clinically important subsets of NSCLC characterized by unique biological behavior and historically limited treatment options. Although these alterations account for a relatively small proportion of NSCLC cases, their identification has become increasingly relevant due to advances in molecular diagnostics and the development of novel targeted therapies. This review provides a comprehensive overview of the epidemiology, molecular biology, and clinical characteristics of *EGFR* exon 20 insertion-mutated and *HER2*-deregulated NSCLC. We discuss current diagnostic approaches, including the role of next-generation sequencing and biomarker testing, and summarize the evolving therapeutic landscape encompassing conventional chemotherapy, immunotherapy, and targeted agents. Particular attention is given to recently approved therapies and emerging treatment strategies, including tyrosine kinase inhibitors and antibody-based therapies that have demonstrated clinically meaningful activity in these patient populations. We also address the major challenges associated with treatment resistance, molecular heterogeneity, and optimal therapeutic sequencing. Finally, we highlight ongoing research efforts and future perspectives aimed at improving outcomes for patients with these rare but clinically significant molecular subtypes of NSCLC.

## Simple Summary

Non–small cell lung cancer (NSCLC) is a heterogeneous disease characterized by multiple molecular alterations that influence prognosis and therapeutic strategies. Among these, epidermal growth factor receptor (*EGFR*) exon 20 insertion mutations and *HER2* dysregulation represent distinct oncogenic drivers associated with unique biological features and therapeutic challenges. This review summarizes the current knowledge regarding the epidemiology, molecular characteristics, diagnostic approaches, available and emerging therapeutic strategies, and ongoing challenges associated with EGFR exon 20 insertion-mutated and HER2-deregulated NSCLC, highlighting future directions for improving patient outcomes.

## 1. EGFR Exon 20 Insertion Non–Small Cell Lung Cancer

### 1.1. Epidemiology and Diagnostic Tests

Epidermal Growth Factor Receptor exon 20 insertion (*EGFR*ex20ins) occurs in ~2–3% of all non–small cell lung cancer (NSCLC) cases, representing ~10–12% of all cancers with documented *EGFR* mutations [1,2,3,4]. Similar to classical *EGFR* mutations, the *EGFR*ex20ins are found more often in women, nonsmokers, and in those with adenocarcinoma histology, with no clear difference by ethnicity [5], with up to 39% of patients with baseline incidence of brain metastases [6].

Structurally, *EGFR*ex20ins are located within the tyrosine kinase domain of EGFR and represent a highly heterogeneous group of molecular alterations. These insertions are typically characterized as in-frame insertions or duplications of 3–21 base pairs (corresponding to 1–7 amino acids) clustered between amino acid positions 761 and 776. Structurally, these insertions occur either at the C-terminal end of the C-helix (codons 761–766) or, more commonly (~90% of cases), within the loop immediately following the C-helix (codons 767–776). These alterations stabilize the kinase domain in its active conformation by pushing the C-helix inward, thereby promoting constitutive *EGFR* signaling. At the same time, the resulting conformational changes create steric hindrance within the ATP-binding pocket, which markedly reduces the binding affinity of conventional EGFR tyrosine kinase inhibitors (TKIs) and explains the intrinsic resistance observed with first-, second-, and many third-generation EGFR TKIs. An important exception includes insertions involving codons 764–765, which appear to preserve a kinase conformation more similar to classical sensitizing *EGFR* mutations and may therefore retain sensitivity to certain EGFR TKIs [7,8,9]. Similarly, most *EGFR*ex20ins remain relatively resistant to osimertinib, a third-generation EGFR TKI [10,11,12,13], and clinically meaningful inhibition with osimertinib often requires drug concentrations higher than those achievable with standard therapeutic dosing. In addition, the therapeutic window for osimertinib dose escalation is limited by off-target inhibition of wild-type EGFR, which contributes to toxicity. Consequently, the prognosis of patients with *EGFR*ex20ins NSCLC has historically been poor, as platinum-based chemotherapy represented the only standard first-line treatment option for many years. Chemotherapy reaches a median progression-free survival (PFS) and overall survival (OS) of 6.6 months and 17.4 months, respectively, and fewer than 10% of patients are alive at 5 years [14,15]. Collectively, these data highlight the urgent need for more effective and molecularly tailored therapeutic strategies in this particularly difficult-to-treat patient population.

Finally, given the extensive molecular heterogeneity of *EGFR*ex20ins, comprehensive next-generation sequencing (NGS) is essential for accurate detection, as targeted PCR-based assays may fail to identify up to 50% of rare insertion variants [16,17]. Almost all *EGFR*ex20ins are mutually exclusive with other canonical oncogene driver alterations; however, co-mutations are relatively common, particularly TP53 co-mutation, which may occur in up to 65% of cases [2,18], as well as *EGFR* amplifications reported in up to 22% of tumors [2]. Additional co-alterations include cyclin-dependent kinase inhibitor 2A and 2B (CDKN2A and CDKN2B, 22% and 16%, respectively), *NK2* homeobox 1 (14%), RB transcriptional co-repressor 1 (*RB1*, 11%) [2]. Therefore, NGS should be considered the cornerstone technique to perform genomic profiling.

### 1.2. From the Past to the Current Standard of Care in First-Line Setting

Previous tyrosine kinase inhibitors (TKI) targeting *EGFR*ex20ins, such as poziotinib, demonstrated initial activity in small phase II trials [19] but failed to confirm efficacy in larger studies [20]. In addition, substantial gastrointestinal toxicity may lead to dose interruptions and reductions, which may negatively impact overall treatment efficacy [20].

A similar pattern was observed with mobocertinib, an irreversible oral TKI targeting *EGFR* and *HER2* exon 20 insertions. In a phase I/II trial, mobocertinib demonstrated clinically meaningful benefit in 114 patients with previously treated *EGFR*ex20ins-positive metastatic NSCLC [21]. The study reported an objective response rate (ORR) of 28% and median PFS of 7.3 months by independent review, and a median OS of 24 months. However, the phase III EXCLAIM-2 trial, which randomized 354 patients to mobocertinib (160 mg daily; n = 179) or chemotherapy (n = 175), did not meet its primary endpoint, reporting an identical median PFS of 9.6 months in both arms (hazard ratio, HR: 1.04, 95% confidence interval, CI: 0.77–1.39; *p* = 0.803). ORRs were also similar between arms (32% vs. 30%, respectively) [22]. Sixty-three patients crossed over from chemotherapy to mobocertinib. In this subgroup, median PFS was 6.8 months with second-line mobocertinib, with no differences in OS (HR: 0.98, 95%CI: 0.62–1.54). Grade ≥ 3 treatment-related adverse events (TRAEs) occurred in 46% and 37% of patients, leading to dose reductions in 45% and 20% of patients, respectively. The reasons underlying the negative results of the EXCLAIM2 trial remain unclear. However, the chemotherapy arm achieved a longer median PFS compared with historical data, while the higher frequency of dose reductions in the mobocertinib arm may also have negatively affected the overall trial outcomes.

In contrast, the promising activity initially observed with amivantamab (an EGFR-MET bispecific antibody) in early-phase studies was subsequently confirmed in a pivotal phase 3 trial. In the phase 1 CHRYSALIS trial, amivantamab was evaluated in 81 patients with pre-treated, metastatic NSCLC harboring *EGFR*ex20ins. The study reported an ORR of 40%, with a median duration of response (DoR) of 11.1 months and median PFS and OS of 8.3 months and 22.8 months, respectively. Grade ≥ 3 adverse events (AEs) occurred in 35% of patients, while grade ≥ 3 TRAEs were reported in 16% of patients, leading to dose reductions and treatment discontinuations in 13% and 4% of patients, respectively [23]. Long-term safety and efficacy data from the CHRYSALIS trial reported similar outcomes [24], and real-world data (RWD) confirmed the improved outcomes with amivantamab compared with external controls in patients with post-platinum, metastatic, *EGFR*ex20ins NSCLC [25]. In addition, emerging data supported the combination of amivantamab and chemotherapy [26], globally providing the rationale for evaluating this combination in the first-line setting.

In the phase 3 PAPILLON trial [27], 308 treatment-naive patients with metastatic *EGFR*ex20ins NSCLC were randomized (1:1) to receive amivantamab plus carboplatin–pemetrexed or chemotherapy alone. The amivantamab arm significantly improved the primary endpoint of PFS compared with chemotherapy alone (median, 11.4 vs. 6.7 months; HR: 0.40; 95% CI: 0.30–0.53, *p* < 0.001), with an ORR of 73% versus 47%, respectively. The OS data were immature at the time of reporting, with a non-significant trend toward survival for the amivantamab arm (HR:0.67; 95%CI: 0.42–1.09; *p* = 0.11), despite 66% of patients in the control arm receiving subsequent amivantamab upon disease progression. Grade ≥ 3 treatment-emergent adverse events (TEAEs) occurred in 75% of patients receiving amivantamab plus chemotherapy and in 53% of those receiving chemotherapy alone, leading to treatment discontinuation of any agent in 24% and 10% of patients, respectively. Amivantamab induced 42% of infusion reactions (1% grade ≥ 3), and other grade ≥ 3 TEAEs, EGFR- and MET-related, included rash (11%), paronychia (7%), and peripheral edema (1%) [27]. In the PAPILLON trial, the incidence of venous thromboembolic events (VTEs) (pulmonary embolism: 7.9% versus 4.5% [grade 3: 2.6% vs. 2.6%]; venous thromboembolism: 6.6% versus 1.9% in amivantamab plus chemotherapy and chemotherapy alone arms, respectively) was consistent with the overall thromboembolic risk typically observed in patients with NSCLC [28]. The PAPILLON trial included up to 23% of patients with treated brain metastases; however, the intracranial activity of amivantamab in *EGFR*ex20ins NSCLC remains incompletely characterized. Nevertheless, in the pivotal trial, a lower incidence of brain progression was observed in the amivantamab-containing arm compared with the chemotherapy-alone arm (5.2% vs. 9.0%), suggesting a potential protective effect against intracranial disease progression [29]. Based on these data, amivantamab was approved by both FDA and EMA in 2024 for first-line treatment of patients with advanced *EGFR*ex20ins NSCLC.

Given the toxicity profile of intravenous amivantamab, which requires intensive premedication with corticosteroids to mitigate infusion-related reactions [30] and close dermatological monitoring with supportive care to manage cutaneous toxicities [31], a subcutaneous formulation has been developed aiming to reduce treatment-related toxicity and improve tolerability. PALOMA-2 (NCT05498428) is a phase 2 bridging study evaluating subcutaneous amivantamab-based regimens in various *EGFR*-mutated advanced NSCLC settings, including 66 treatment-naive patients with *EGFR*ex20ins receiving subcutaneous amivantamab in combination with pemetrexed-carboplatin chemotherapy [32]. The confirmed ORR was 71%, with a median DoR of 10.6 months and a median PFS of 12.2 months. Subcutaneous administration reduced the infusion-related reactions to 6%, with no grade ≥ 3 reactions. The subcutaneous formulation primarily reduced the risk of infusion-related reactions; however, the rates of grade ≥ 3 cutaneous toxicities, including rash (9%) and paronychia (5%), remained comparable to those observed with intravenous administration. Treatment discontinuation due to TRAEs occurred in 12% of participants receiving subcutaneous amivantamab. Overall, the activity of subcutaneous amivantamab in this setting is consistent with that reported for intravenous amivantamab in the pivotal phase 3 trial [27].

Although the subcutaneous formulation may improve patient convenience, its benefit appears largely limited to reducing infusion-related reactions, without meaningfully mitigating other on-target toxicities. Alternative dosing strategies, such as dose optimization or extended dosing intervals based on the half-life of amivantamab (11.3 ± 4.53 days) [33], may be challenging to implement in clinical practice but remain of interest as potential approaches to improve tolerability and treatment adherence. In addition, defining both primary resistance and mechanism of acquired resistance to amivantamab is clinically relevant to inform subsequent treatment strategies in *EGFR*ex20ins NSCLC. In a small cohort analysis in liquid biopsies, baseline *TP53* co-mutation did not show a clear correlation with primary resistance to amivantamab; however, *EGFR* amplification was associated with significantly shorter PFS compared with tumors without amplification [18], suggesting that specific genomic contexts may influence therapeutic durability. Additionally, several acquired resistance mechanisms to amivantamab were reported involving *EGFR*-dependent or *EGFR*-independent pathways but also broader changes in tumor biology that promote adaptability and survival under treatment pressure [18].

### 1.3. New Targeted Therapies for EGFR Exon 20 Insertions

Despite the modest clinical benefit and increased toxicity reported by poziotinib [20] and mobocertinib [22], new TKIs have been developed for this subset of lung adenocarcinomas.

#### 1.3.1. Sunvozertinib

In patients with metastatic, platinum-refractory *EGFR*ex20ins NSCLC, sunvozertinib has been tested in two phase II clinical trials. The WU-KONG 6 trial [34] was conducted only in China and evaluated sunvozertinib (300 mg daily) in 104 patients. The confirmed ORR was 61%, and the DoR was 8.3 months, with benefit in all subgroups, leading to conditional approval of this drug in China. The second trial, the multinational pivotal WU-KONG1B [35], evaluated sunvozertinib at the dose of 200 mg (n = 91) or 300 mg (n = 101) daily. In the trial, 35% of patients were from non-Asian countries, 24% of patients had baseline brain metastasis, and approximately 14% had been previously treated with amivantamab. The confirmed ORR (cORR) was identical at both doses, 46%. However, higher cORRs were observed at the dose of 300 mg versus 200 mg in non-Asian patients (43.3% vs. 33.3%), among those with baseline brain metastasis (52.4% vs. 28.6%), and in those with previous amivantamab treatment (41.7% vs. 25%). The DoR was 11.1 months and 13.8 months at the two different doses, whereas the median PFS was 8.4 months and 6.9 months at the 200 and 300 mg doses, respectively, with 1-year OS rates of 62% and 69%, respectively. The incidence of grade ≥ 3 TRAEs was generally higher at 300 mg than 200 mg once daily (58.6% and 40.7), leading to a higher incidence of dose reductions (38.7% vs. 23%) and treatment discontinuations (7.2% vs. 4.4%). In a post hoc analysis in patients with measurable intracranial lesions (n = 5 at 200 mg; n = 10 at 300 mg), the intracranial ORRs were 0% and 40%, respectively. Based on the overall efficacy results, in July 2025 the FDA granted accelerated approval to sunvozertinib at the dose of 200 mg per day in pre-treated patients with metastatic *EGFR*ex20ins NSCLC.

In treatment-naive patients with metastatic *EGFR*ex20ins NSCLC, sunvozertinib (n = 28) has also reported clinically meaningful results with a confirmed ORR of 71.4%, and estimated median PFS at 300 mg of 12.4 months [36].

Finally, the phase 3 WU-KONG28 trial has been conducted in the first-line setting [37]. The trial randomized 324 patients to receive sunvozertinib (300 mg daily) or platinum-pemetrexed chemotherapy. The trial reached the primary endpoint as sunvozertinib significantly improved the PFS compared with chemotherapy (10.3 versus 7.5 months; HR: 0.65; 95% CI: 0.50–0.85; *p* < 0.001). The ORR was 58.9% and 31.1% in the sunvozertinib and chemotherapy arms, respectively, with a median DoR of 11.2 and 7.1 months, respectively. Grade ≥ 3 TRAEs occurred in 61.3% in the TKI arm versus 49.3% in the chemotherapy arm, leading to treatment dose reductions in 41% and 24%, respectively, and discontinuations in 7.4% and 11.3%. The most common AEs in the sunvozertinib arm were diarrhea (87%, grade ≥ 3 in 14%) and rash (53%, grade ≥ 3 in 0.6%). OS is still immature, with no difference between the two arms; however, 90.2% of patients in the chemotherapy arm crossed over to sunvozertinib at the time of disease progression. These data support sunvozertinib as a novel first-line strategy in *EGFR*ex20ins NSCLC, but it remains unknown whether a 200 mg dose could achieve similar clinical outcomes with a better safety profile.

#### 1.3.2. Firmonertinib

Firmonertinib is an oral, highly brain-permeable, selective EGFR TKI with broad efficacy and selectivity against *EGFR* mutations. The drug has been approved in China for the treatment of patients with advanced NSCLC harboring common sensitizing *EGFR* T790M mutations.

In the phase Ib FARVOUR trial [38], firmonertinib demonstrated promising efficacy in patients with NSCLC with *EGFR*ex20ins in both treatment-naïve and previously treated settings. Among treatment-naive patients receiving firmonertinib 240 mg daily (n = 30), the ORR was 78.6%, and the median DoR was 15.2 months. In previously treated patients, ORRs were 46% and 39% at doses of 240 mg (n = 30) and 160 mg (n = 30), respectively, with median DoR of 13.1 and 9.7 months. Grade ≥ 3 TRAEs occurred in 13% of treatment-naive patients, leading to a dose reduction in 13% of cases but no treatment discontinuations. Among previously treated patients, grade ≥ 3 TRAEs occurred in 28% and 19% of patients treated with 240 and 160 mg, respectively, resulting in dose reductions in 18% and 11% of cases, and treatment discontinuations in 4% of patients at both dose levels. The activity of firmonertinib 240 mg once daily in previously treated metastatic *EGFR*ex20ins NSCLC was subsequently confirmed in the pivotal phase II FURMO-003 trial, which enrolled 71 patients previously treated with platinum-based chemotherapy and included 23% of patients with baseline brain metastases. The cORR was 44.3%, with a median DoR of 8.3 months, and median PFS and OS of 8.3 months and 21.2 months, respectively, without new safety data [39].

**Figure 1:**
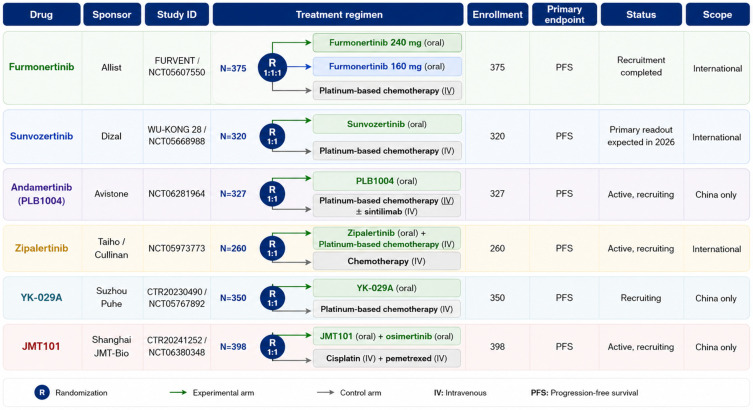
Clinical trials in metastatic non–small cell lung cancer harboring *EGFR* exon 20 insertions. IV: intravenous.

Based on these data, the phase 3 FURVENT/FURMO-004 trial (NCT05607550), evaluating furmonertinib (160 mg and 240 mg) versus chemotherapy in patients with metastatic previously untreated *EGFR*ex20ins NSCLC has been launched. The enrollment of the trial has been completed (Figure 1). Furmonertinib is also being studied in an ongoing global phase 1b clinical trial in NSCLC patients with other uncommon EGFR activating or HER2 mutations (FURMO-002; NCT05364073).

#### 1.3.3. Zipalertinib

Zipalertinib is an irreversible oral EGFR TKI designed to selectively target EGFRex20ins while sparing wild-type EGFR. It has a distinct pyridopyrimidine scaffold, which differentiates it from other ex20ins TKIs, and shows potent inhibition of EGFR signaling and tumor cell growth with reduced off-target activity, including minimal inhibition of HER2 [40].

The REZILIENT 1 is a phase I/II trial evaluating zipalertinib in patients with recurrent or metastatic NSCLC harboring *EGFR*ex20ins. Among 73 previously treated patients, zipalertinib reported encouraging antitumor activity (ORR 38.4%, median DoR of 10 months, and median PFS of 10 months) and safety (grade ≥ 3 TRAEs of 23%, with dose reductions and treatment discontinuation in 14% and 8% of patients, respectively). Results of the trial confirmed zipalertinib 100 mg twice daily as the recommended dose for further clinical development [40].

Later on, 244 previously treated patients (n = 143 previous chemotherapy only, and n = 101 who had also received previous *EGFR*ex20ins-directed therapy) were enrolled and treated with zipalertinib 100 mg twice daily [40]. In the overall population, 42% of patients had brain metastases, 34% had received previous amivantamab, and 66% of patients were non-Asian. In the efficacy population (n = 176), the ORR was 35.2%, and the median DoR was 8.8 months. Similar data were reported in patients who had received prior platinum-based chemotherapy without *EGFR*ex20ins-targeted therapy: ORR was 40%, median DoR of 8.8 months, and median PFS of 8.8 months. The safety profile mirrored previous data with 29.5% of grade ≥ 3 TRAES, and dose reduction and treatment discontinuation in 14.3% and 8.2% of patients overall. Zipalertinib (100 mg twice daily) was evaluated in 84 patients previously treated with platinum-based chemotherapy and *EGFR*ex20ins–targeted therapies. This population included 54 patients who had received prior amivantamab only and 30 patients who had received amivantamab in combination with other *EGFR*ex20ins-directed targeted therapies. In these respective subgroups, the ORR was 31.5% and 20%, the median DoR was 9.5 months and 8.3 months, and the median PFS was 7.4 months and 5.4 months, respectively [41].

The phase II REZILIENT2 study evaluated intracranial activity of zipalertinib at the recommended doses [42]. Zipalertinib demonstrated clinically meaningful intracranial antitumor activity, with a 31.3% intracranial ORR and 68.8% intracranial disease control rate by RANO-BM criteria in patients with NSCLC harboring *EGFR*ex20ins or other uncommon single or compound mutations.

Finally, the safety and efficacy of first-line zipalertinib is being investigated in combination with standard platinum-based chemotherapy in patients with locally advanced or metastatic *EGFR*ex20ins NSCLC in the phase III REZILIENT3 trial (NCT05973773, Figure 1).

#### 1.3.4. Other New Targeted Therapies for *EGFR*ex20ins NSCLC

YK-029A is a third-generation EGFR TKI, and the preliminary efficacy of YK-029A (200 mg once per day) in 26 treatment-naive patients with *EGFR*ex20ins reported an ORR of 73%, with a median DoR and PFS of 7.5 months and 9.3 months, respectively [43]. Based on these results, there is an ongoing phase 3 trial in the first-line setting (NCT05767892, Figure 1).

The phase 2 KANNON study evaluating andamertinib (240 mg/day), a selective and irreversible tyrosine kinase inhibitor, in 94 pre-treated advanced *EGFR*ex20ins NSCLC reported an ORR of 43% and median DoR of 8.7 months. The median PFS was 6.2 months, and the 1-year OS rate was 71%. The incidence of grade ≥ 3 TRAEs was 40.2% [44]. In the meantime, andamertinib is currently under investigation in the first-line setting in a pivotal phase III trial (NCT06281964, Figure 1).

The phase II BECOME study, evaluating becotarug (JMT101, a humanized IgG1 monoclonal antibody targeting EGFR, 6 mg/kg every 2 weeks) plus osimertinib (160 mg/day), demonstrated encouraging and clinically meaningful outcomes (ORR 50%, median PFS of 6.9 months, and median OS of 18.0 months) in 112 platinum-pretreated *EGFR*ex20ins-positive NSCLC [45]. A phase 3 study of becotarug plus osimertinib in treatment-naïve *EGFR*ex20ins metastatic NSCLC is ongoing (NCT06380348, Figure 1).

Enozertinib (ORIC-114), a highly selective, brain penetrant EGFR and HER2 inhibitor, has reported activity (at the dose of 80 mg or 120 mg) in second-line as well as in treatment-naïve patients with metastatic *EGFR*ex20ins NSCLC [46]. The preliminary ORR in second-line patients who were evaluable for efficacy was 45% (80 mg: n = 20) and 20% (120 mg: n = 15). In the first-line patients’ cohort, only the 120 mg dose level had sufficient evaluable patients to assess efficacy (n = 15), yielding an ORR of 67%; however, 73% of patients had dose reductions down to 80 mg. Intracranial ORR was 71% in seven patients treated in the first-line cohort. Data from this trial support 80 mg once daily as the likely recommended dose for further development.

Other agents with clinical or preclinical data in *EGFR*ex20ins include: BEBT-109 [47], STX-721 [48].

### 1.4. Current and Future Challenges

#### 1.4.1. Activity of Targeted Therapies According to *EGFR* Exon 20 Insertion Subtype

*EGFR*ex20ins is a highly heterogeneous disease, with more than 100 unique variants identified. Indeed, exon 20 loop insertions can be separated into two distinct groups: near-loop (codons 767 to 772) and far-loop (codons 773 to 776) insertions, which may define divergent and sometimes variant-specific responses to targeted therapies [49]. The majority of TKIs are more active in near-loop insertions compared to far-loop insertions, including: poziotinib (ORR: 25.9% vs. 9.8%, *p* = 0.004, for near- vs. far-loop insertions, including 66 and 19 patients, respectively) [20], zipalertinib (ORR: 41.5% vs. 22%, respectively, with 52 and 9 patients in each group) [40], and sunvozertinib. In the phase 2 WU-KONG1B trial [35] and according to the two doses evaluated of sunvozertinib (200 mg and 300 mg daily), the ORRs were higher in insertions near the loop (61% and 72%), far from the loop (32% and 21%), and C-helix (1.2% and 2.8%). In contrast, comparable efficacy for both near- and far-loop insertions has been reported with mobocertinib [20] and YK-029A [43]. Finally, in the phase I CHRYSALIS trial, amivantamab reported an ORR of 41% and 25% in near-loop (n = 54) and far-loop (n = 8), respectively [23].

Taken together, the data show that *EGFR*ex20ins NSCLC are a heterogeneous population of mutations, and that insertion location alters TKI binding, modifying treatment efficacy. Nevertheless, owing to the small sample size, the nature of subgroup and exploratory analyses, future studies with a larger number of patients are needed to corroborate this correlation. However, these data emphasize the future interest of personalizing TKIs to patients’ tumor mutation subtype. Indeed, it remains challenging to know whether first-line treatment options should be intensified for those patients whose tumors harbor *EGFR*ex20ins far-loop, rather than treatment with a TKI monotherapy. Although data from the WU-KONG28 trial [37] suggest benefit of the monotherapy in near and far-loop, more data is necessary to confirm this observation.

#### 1.4.2. Optimal First-Line Treatment Approach

Despite the limitations of cross-trial comparisons, most of the newer TKIs for *EGFR*ex20ins tumors [35,38,40,44] have reported broadly similar activity in pre-treated patients with ORR of approximately 40%, median PFS of 8 months, and more than 60% of patients alive at 12 months, suggesting a plateau in the clinical benefit in this setting. Interestingly, some of these agents, including sunvozertinib [35] and zipalertinib [41], have demonstrated activity in patients previously treated with amivantamab, suggesting the potential feasibility of a sequential treatment strategy, although the small sample sizes preclude definitive conclusions. However, all these TKI agents had consistently reported higher efficacy in treatment-naive patients than in pre-treated populations. This observation has been confirmed in the phase 3 WU-KONG28 trial supporting the superiority of upfront sunvozertinib over chemotherapy [37]. Despite not being directly compared with the current standard in this setting (amivantamab plus chemotherapy), considering the oral administration, potential intracranial activity, and potentially more favorable toxicity profiles, they are more attractive as the preferred treatment option in first-line in the future. Despite these clinically meaningful results, the efficacy of *EGFR*ex20ins TKI in the first-line setting still appears more modest than that achieved with TKI targeting common *EGFR* mutations. The trial also raises questions regarding the optimal dosing of *EGFR*ex20ins TKIs to minimize toxicity that may negatively affect patients’ quality of life. Importantly, persistent grade 2 diarrhea or rash may substantially impair daily activities; therefore, not only the grade but also the duration of these low-grade TRAEs should be systematically reported.

All pivotal first-line phase 3 trials of these *EGFR*ex20ins TKIs use chemotherapy as the control arm rather than amivantamab plus chemotherapy, which may complicate direct positioning within current treatment algorithms. Another key challenge should these TKIs become the future first-line standard is determining whether they should be used as monotherapy or in combination with chemotherapy, as has been established in classical *EGFR*-mutant metastatic NSCLC where TKI–chemotherapy combinations improve PFS and OS compared with TKI alone [50,51]. In *EGFR*ex20ins, only zipalertinib is currently being evaluated in first-line in combination with chemotherapy; therefore, only robust prospective data—or potentially strong cross-trial comparisons demonstrating a clinically meaningful improvement in outcomes with the combination instead of monotherapy—will clarify whether combination strategies should become the new standard of care. In the future, several factors like the occurrence of baseline brain metastases, mutation subtype, co-occurring genomic alterations, tumor burden, and treatment access will be factors that can guide the optimal first-line treatment option for each patient.

Finally, increasing the knowledge regarding the mechanisms of primary and acquired resistance is challenging as it may help to elucidate the potential subsequent treatments. However, data regarding mechanisms of acquired resistance to currently available agents remains limited [18,52].

#### 1.4.3. Treatment of *EGFR* Exon 20 Insertions in Early-Stage Setting

**Figure 2:**
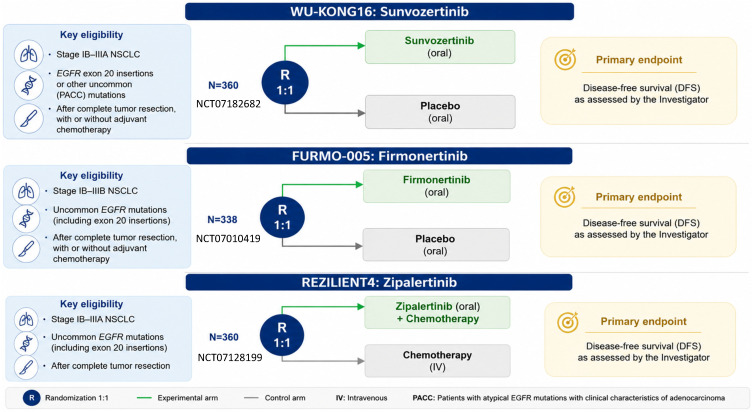
Clinical trials in the adjuvant setting of non–small cell lung cancer harboring *EGFR* exon 20 insertions.

Based on the role of EGFR TKI in common sensitizing *EGFR*-mutant tumors [53] and that early-stage *EGFR*ex20ins NSCLC may have a poorer prognosis and cumulative incidence of brain metastases at 5 years of 19% [54], it seems relevant to explore *EGFR*ex20ins TKI in the early-stage setting. Several phase III trials are ongoing (Figure 2). The WU-KONG16 study is assessing adjuvant sunvozertinib versus placebo in patients with stage IB–IIIA NSCLC harboring *EGFR*ex20ins or other uncommon *EGFR* mutations after complete surgical resection, with or without adjuvant chemotherapy, using disease-free survival (DFS) as the primary endpoint. Similarly, the FURMO-005 trial is investigating firmonertinib in the adjuvant setting for patients with resected stage IB–IIIB NSCLC with uncommon *EGFR* mutations, also comparing against placebo with investigator-assessed DFS as the primary outcome. In contrast, the REZILIENT4 study is evaluating Zipalertinib in combination with chemotherapy versus chemotherapy alone in patients with resected stage IB–IIIA disease, with DFS as the primary endpoint, thereby exploring the potential added benefit of targeted therapy alongside standard adjuvant treatment.

#### 1.4.4. Antibody–Drug Conjugates in *EGFR* Exon 20 Insertions

In patients with common sensitizing *EGFR*-mutant NSCLC and previously treated with EGFR TKI, with or without chemotherapy, antibody–drug conjugates (ADCs) targeting TROP2 [55,56,57] have demonstrated clinically meaningful activity, and some agents such as datopotamab deruxtecan [57] have received FDA approval for patients with EGFR-mutant NSCLC after progression on both TKI and platinum-based chemotherapy. In a phase 2 trial, sacituzumab tirumotecan, an anti-TROP2 ADC, was evaluated in 19 patients with pre-treated *EGFR*ex20ins metastatic NSCLC, reporting an ORR of 37% and a median PFS of 9.0 months, with grade ≥ 3 TRAEs in 52.4% of patients [58]. Similarly, the izalontamab brengitecan, a bispecific ADC simultaneously targeting EGFR and HER3, reported an ORR of 86% in the same setting, with a median PFS of 10.2 months [59]. Finally, patritumab deruxtecan, an anti-HER3 ADC, has demonstrated more modest activity in this setting, with an ORR of 27.7% and a median PFS of 5.5 months [60]. Although these trials have not yet reported intracranial activity in NSCLC harboring genomic alterations, collectively they suggest a potential role of ADCs in the sequential treatment landscape of *EGFR*ex20ins NSCLC, which warrants further validation in ongoing studies. Indeed, exploring combination strategies involving antibody–drug conjugates (ADCs) and tyrosine kinase inhibitors (TKIs) with demonstrated activity in *EGFR*ex20ins NSCLC warrants further clinical investigation.

## 2. HER2-Deregulated Non–Small Cell Lung Cancers

### 2.1. Epidemiology and Diagnostic Testing

HER2 is a receptor tyrosine kinase belonging to the ErbB family (*ERBB2* gene), without a binding domain, which dimerizes with ERBB2 (homodimerization) as well as with other ERBB family receptors (heterodimerization), leading to activation of mitogen-activated protein kinase (*MAPK*) and phosphatidylinositol-3 kinase (*PI3K*) pathways in a kinase-dependent manner.

*HER2* dysregulation, including gene mutations, amplifications, and protein overexpression, occurs in 5% of all NSCLC and promotes oncogenesis. *HER2* dysregulation [61].

Activating *HER2* mutations occur in 2–4% of NSCLC [62,63]. Up to 70% of them are insertions in exon 20, within the TKD, the A775_G776insYVMA representing up to 60% of all *HER2* insertions, followed in prevalence by G776delinsVC/LC/VV/IC (10–20%) [61,64]. Activating point mutations can also occur in exon 20 [61] or in the extracellular domain [64], such as S310F/Y point mutations in the extracellular domain occurring in up to 10% of all NSCLC with *HER2* mutations. Other point mutations in the tyrosine kinase, in the juxta- or transmembrane domains, are less common [65]. Patients with oncogenic TKD and non-TKD mutations appeared to have different demographic and clinical characteristics. Patients with TKD mutations are younger at diagnosis (<65 years at diagnosis: TKD vs. non-TKD: 40.6% versus 26.2%), commonly females (64.0% versus 52.3%), never-smokers (51.8% versus 21.5%), and have adenocarcinoma histology (97.0% versus 75.4%) than patients with non-TKD mutations [63,66]. Of note, the frequency of brain metastases was comparable in tumors with *HER2* mutations in TKD versus non-TKD (21.3% and 15.4%, respectively) [63].

*HER2* mutations can be detected by polymerase chain reaction (PCR) or NGS [67]. Similarly to what has been shown for *EGFR* exon20ins [68,69], NGS might show higher sensitivity and allow the identification of less common variants. Concurrent *HER2* amplification was more common in patients with TKD mutations (12.7%) than in those with non-TKD mutations (3.1%). Patients with TKD mutations had significantly fewer co-mutations than patients with non-TKD mutations, including *EGFR* (3.6% vs. 18.5%; *p* = 0.0003), *KRAS* (4.6% vs. 15.4%; *p* = 0.01), and *PIK3CA* mutations (3.6% vs. 12.3%; *p* = 0.01), and both subgroups had *TP53* co-mutations in up to 50% of cases. Finally, the immunogenic profile is different, as the occurrence of TMB ≥ 10 mutations/megabase for TKD patients was 18.3% versus 62.2% for non–TKD-mutant patients (*p* < 0.0001) [63].

*HER2* gene amplifications have been reported in less than 2% of NSCLC, and only a small percentage coexist with *HER2* activating mutations (approximately 3–12%) [61,63]. *HER2* gene amplification occurs more frequently in male patients with a smoking history, and in non-adenocarcinoma histology [61,63,65,70].

*HER2* gene amplifications are detected by fluorescence in situ hybridization (FISH) or NGS, the latter allowing the evaluation of both point mutations and copy number variations [62]. *HER2* gene amplification is not mutually exclusive with other oncogenic drivers, such as *EGFR* or *KRAS* mutations [71], and it might also emerge as a mechanism of acquired resistance under treatment with EGFR TKIs in *EGFR*-mutated NSCLC [72]. Although *HER2* gene amplification may induce HER2 protein expression detected by immunohistochemistry (IHC) [65,67,70,73], HER2 may also originate from aneuploidy, which plays a major role in inducing protein overexpression [74]. Therefore, IHC scoring is not used as a screening method for *HER2* gene amplification in NSCLC [62,71].

The prevalence of HER2 overexpression is heterogeneous, ranging from 20% to 40% due to different HER2-IHC scoring [62]. To date, no HER2 IHC scoring criteria have been established specifically for NSCLC [62]. For non-breast cancer tumors including NSCLC, the ASCO/CAP gastric cancer HER2 IHC scoring system is commonly applied [75]. HER2 overexpression is a negative prognostic factor in NSCLC, and it is not mutually exclusive with other oncogenic drivers [62,71]. HER2 overexpression can also emerge as a mechanism of acquired resistance to EGFR TKIs in NSCLC with activating *EGFR* mutations [76,77].

Currently, a personalized treatment approach has been established in *HER2*-mutant NSCLC with TKI and ADC. Similarly, ADC as a monotherapy has been approved for the treatment of adult patients with unresectable or metastatic HER2-positive (immunohistochemistry [IHC] 3+) solid tumors who have received prior treatment and who have no satisfactory treatment options. However, no approved treatment for *HER2* gene-amplified NSCLC.

### 2.2. HER2 Mutations: From the Past to the Current Standard of Care in First-Line Setting

Before specific treatment approaches, the median OS of patients with HER2-mutant NSCLC remained poor [78,79], especially A775_G776YVMA variants, which seemed to be associated with worse prognosis [80]. The median OS was 13.5 months, and among patients receiving first-line chemoimmunotherapy, median OS was 21.1 months in patients with TKD mutations versus 11.7 months in those with non-TKD mutations [63], suggesting that the addition of immunotherapy may improve survival outcomes [64].

Targeting HER2 has been first attempted with monoclonal antibodies: pertuzumab plus trastuzumab achieving an ORR of 21% (95%CI 5–51) in patients with NSCLC harboring *HER2* mutations [81].

#### 2.2.1. Pan-ErbB Inhibitors and Mobocertinib

Pan-ErbB inhibitors are TKIs targeting multiple products of the ERBB gene family, including HER2 [82]. Since HER2 is activated through dimerization with other proteins belonging to the ErbB family (homodimerization and heterodimerization), pan-ErbB inhibitors, including afatinib [83], neratinib [84], and dacomitinib [85], have been investigated in *HER2*-mutated NSCLC, with unsatisfactory results (ORR ranging between 4% and 12% in pre-treated patients).

In a phase II multicohort trial (n = 30), poziotinib at the dose of 16 mg once daily reported an ORR of 27% and median PFS of 5.5 months [86]. Most patients (n = 23, 93%) were pre-treated, with previous treatment lines including the pan-ErbB kinase inhibitors afatinib (n = 5, 15%) and tarloxotinib (n = 1, 3%), and the anti-HER2 monoclonal antibodies trastuzumab (n = 3, 9%) and pertuzumab (n = 1, 3%) [86]. The multicentric ZENITH20 phase II trial showed similar outcomes in pre-treated patients (n = 90), with an ORR of 27.8%, and median PFS of 5.5 months [87]. In the small subgroup (n = 7) of G778_P780dupGSP mutations, ORR reached 100% [87]. In the subgroups of treatment-naïve patients (n = 80), the ORR was 39% [88].

In pre-treated patients, a phase II study with pyrotinib (400 mg once daily) reported an ORR of 30% with median PFS and OS of 6.9 and 14.4, respectively [89]. Of note, the activity of pyrotinib was similar in the first-line setting (n = 28, ORR: 35.7%, with median PFS and OS of 7.3 and 14.3 months, respectively) [90].

The major limitation of all these unselective agents, apart from limited clinically meaningful benefit, was the toxicity profile due to the concomitant inhibition of the EGFR pathway [86,87,88]. Severe toxicities include: grade ≥ 3 skin rash (≈45–50%), paronychia (≈20%), stomatitis (≈20–25%) and diarrhea (20–25%) [86,87,88].

Finally, preclinical data suggest mobocertinib could be effective against certain non-YVMA insertions [91], but clinical data remain limited, with only one phase I trial of 28 patients treated with mobocertinib (120 or 80 mg/die) plus T-DM1 (3.6 mg/kg every 3 weeks) [92].

#### 2.2.2. Sevabertinib

Sevabertinib (BAY 2927088) is a reversible inhibitor of both *EGFR* and *HER2* exon 20 insertions [93]. In the phase I/II SOHO-01 study, 209 patients received sevabertinib at the expansion dose of 20 mg/day in cohort D (previously treated, no previous *HER2* ex20ins-targeted therapy, n = 81); cohort E (previously treated with HER2-targeted ADCs, n = 55); cohort F (previously untreated, n = 73). Asian ethnicity was the most represented one (70%, 58%, 70% of patients in cohort D, E, and F, respectively). The Y772_A775dupYVMA was the most represented insertion (60%, 73%, 79% in cohort D, E, and F, respectively). The ORR was 64% in cohort D, 38% in cohort E, and 71% in cohort F of previously untreated patients [94]. Median PFS was 8.3 and 5.5 months in cohorts D and E, respectively, being immature for cohort F [94]. The most common grade ≥ 3 TRAE was diarrhea, occurring in 19 (23%), 6 (11%), and 4 (5%) cases in cohorts D, E, and F and in one 1 (3%) patient treated with sevabertinib 10 mg/die in cohort D1 [94].

**Figure 3:**
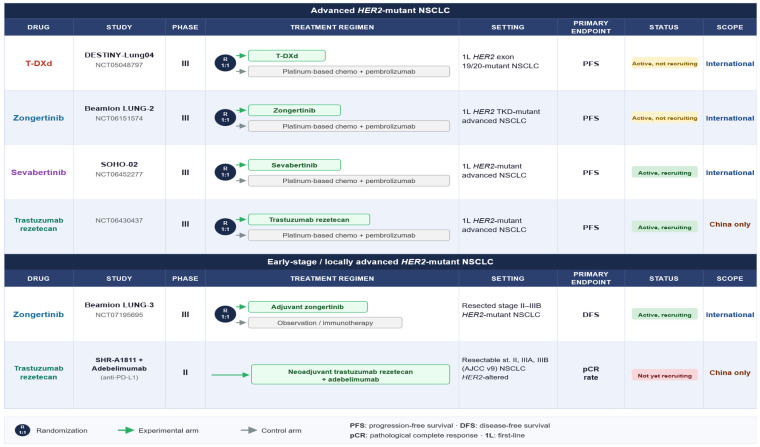
Clinical trials in HER2 mutant non–small cell lung cancer across stages of the disease.

Based on this data from cohort D, in 2025 the FDA granted accelerated approval to sevabertinib for patients with non-squamous NSCLC with *HER2* TKD activating mutations who had received previous treatment. Indeed, based on the promising data in first-line, the phase III SOHO-02 trial (NCT06452277) is ongoing and comparing sevabertinib (20 mg/day) to chemotherapy with or without pembrolizumab as first-line treatment in patients with previously untreated NSCLC with *HER2* activating mutations (Figure 3) [95].

#### 2.2.3. Zongertinib

Zongertinib is an oral, irreversible HER2 TKI, which spares the EGFR domain. The global, multicohort phase I Beamion LUNG-1 trial investigated zongertinib in *HER2*-altered advanced or metastatic solid tumors in the phase Ia and in advanced or metastatic *HER2*-mutant NSCLC in the phase Ib [96].

In cohort 1, 75 previously treated patients whose tumors harbor a TKD mutation received zongertinib at a dose of 120 mg. The ORR reached 71% (including an ORR of 81% in the A775_G776insYVMA insertion, and lower ORR in other insertion subtypes) and the median PFS was 12.4 months, with grade ≥ 3 TRAEs of 17%, with dose reduction and discontinuation in 7% and 3% of patients, respectively [96]. Based on these data, the FDA granted accelerated approval of zongertinib in this setting in 2025 for patients with previously treated NSCLC with HER2 TKD activating mutations. In contrast, the EMA has not approved, for instance, any HER2 TKI for this population in any setting.

In cohort 5, zongertinib was evaluated among 31 patients with *HER2* mutant tumors previously treated with HER2-directed ADCs and reporting an ORR of 42% and median PFS of 6.8 months [96,97]. This data may suggest the potential sequential approach after ADC therapy for these patients.

In cohort 3, zongertinib was evaluated in 20 patients with previously treated NSCCL with non-TKD *HER2* mutations. The ORR was 30%, and median PFS has not been reached [97], suggesting potential efficacy regardless of the HER2-mutation subtype, although in the non-TKD group outcomes were lower.

In cohort 4, 30 patients with active brain metastases received zongertinib, whether they had or had not received prior systemic treatment (n = 30). Intracranial objective response (RANO-BM criteria) was 47% [98]. In this cohort, eight patients received zongertinib as first-line treatment, and four of them (50%) had intracranial response. The grade ≥ 3 TRAEs in this cohort were 17%.

However, the most clinically impactful data from the phase I Beamion LUNG-1 trial come from cohort 2 in the treatment-naïve population (n = 74). One third of patients had baseline brain metastasis. In this cohort, zongertinib reported an ORR of 77%. To note, confirmed ORR was 84% in the A775_G776insYVMA subgroup (n = 50). However, responses were also observed among patients harboring other TKD mutations (58%). Median PFS was 14.4 months. The incidence of grade ≥ 3 TRAEs was 19%, leading to treatment discontinuation in 9% of cases. Of note, the EGFR-sparing mechanism of action of zongertinib influences its safety profile: cutaneous and gastrointestinal AEs are less frequent, whereas ALT increase (4%) and decreased ejection fraction (4%) were the most common grade ≥ 3 AEs [98]. Therefore, in February 2026, the FDA granted accelerated approval to zongertinib for first-line treatment of non-squamous NSCLC with activating HER2 tyrosine kinase domain mutations, based on data from a Phase I clinical trial, despite the fact that results from the ongoing Phase III BEAMION LUNG-2 (NCT06151574) trial—comparing zongertinib with standard of care in the first-line setting—are still pending after completion of enrollment (Figure 3).

#### 2.2.4. HER2 Mutations—ADCs

The first ADC tested in pre-treated patients with *HER2* mutant NSCLC was ado-trastuzumab emtansine (T-DM1), which conjugates trastuzumab with emtansine (DM1). Among 18 patients, the ORR was 44% with a median PFS of 5 months [99]. However, new ADCs have been included as a potential strategy in this setting.

Trastuzumab deruxtecan (T-DXd) is an anti-HER2 monoclonal antibody linked to a topoisomerase I inhibitor through a tetrapeptide-based cleavable linker. The pivotal phase II DESTINY-Lung01 tested T-DXd at the dose of 6.4 mg/kg in 55 previously treated patients. The ORR was 55% with a median PFS of 8.3 months and median OS of 17.8 months. Benefit occurred regardless of brain metastases status and number of previous lines. However, 28% of patients reported interstitial lung disease (ILD) of any grade [100]. Therefore, the phase II DESTINY-Lung02 trial was launched, which evaluated T-DXd at the doses of 5.4 mg/kg and 6.4 mg/kg every three weeks in the same setting. ORR was 49% and 56%, respectively. The incidence of ILD was 12% at the dose of 5.4 mg/kg and 28% with higher doses (2.0 grade ≥ 3 in each arm) [101]. As the trial confirmed the lower doses did not impact clinical outcomes and may reduce the risk of ILD, both the FDA and the EMA approved T-DXd at the dose of 5.4 mg/kg for *HER2*-mutated NSCLC, after platinum-chemotherapy with or without immunotherapy [66]. Indeed, pooled analyses of the DESTINY-Lung 01&02 trial have reported intracranial activity of T-DXd (n = 41), with an intracranial ORR of 50% at 5.4 mg/kg [102]. However, it remains a small sample size with a heterogeneous population with brain metastases.

Moreover, results from the phase III DESTINY Lung-04 trial (NCT05048797) are awaited. The trial has investigated T-DXd vs. platinum, pemetrexed and pembrolizumab as the first-line treatment for advanced or metastatic *HER2*-mutated NSCLC. Of note, in contrast with clinical trials evaluating HER2 TKI in first line, the DESTINY Lung04 trial is the only one that allows the enrollment of patients with non-TKD *HER2* mutations.

The phase II HORIZON-Lung trial [103] evaluates trastuzumab rezetecan (another anti-HER2 ADC with a topoisomerase I payload) at the dose of 4.8 mg/kg every 3 weeks among 94 Chinese patients with previously treated *HER2*-mutant NSCLC (97% had TKD mutations, including 72% Ala775_Gly776insTyrValMetAla). The drug reported an ORR of 73% and median PFS of 11.5 months, with grade ≥ 3 TRAEs of 23% and ILD (any grade of 7%) [103]. Although this ADC has reported lower ILD than T-DXd (indirect comparisons), the fact that the trial was conducted in a single country may limit the generalizability of these results to global patient populations.

BL-M07D1 is an anti-HER2 ADC linked to a topoisomerase I inhibitor via a cleavable linker. At the dose of 4.4 mg/kg once every, BL-M07D1 achieved an ORR of 56.9% in 58 patients with previously treated NSCLC with *HER2* mutations [104].

Both trastuzumab rezetecan and BL-M07D1 are currently under investigation in phase III trials after proving efficacy in phase II studies (NCT06430437, NCT07178795).

The activity and safety of combination strategies of anti-HER2 ADCs and immunotherapy have also been explored. The phase Ib DS8201-A-U106 study investigated T-DXd plus pembrolizumab in advanced, immunotherapy-naïve, NSCLC harboring *HER2* mutations, or showing HER2 expression (IHC scoring 1+, 2+, 3+). Across all patients (n = 55), interstitial lung disease (ILD)/pneumonitis occurred in 11 (20%) patients. In the *HER2*-mutant cohort, ORR was 66.7% (95% CI, 48.2–82.0), with responses observed in 22 out of 33 patients [105]. In the subset of patients who had received no previous line of treatment, responses were observed in 80% of cases (95%CI, 56.3–94.3), with 10% being complete responses [105].

## 3. HER2 Overexpression

HER2 protein expression was first targeted by monoclonal antibodies, following the therapeutic paradigm established in breast cancer. In 2003, a phase II randomized trial conducted in patients with NSCLC and HER2 positivity (i.e., HER2 IHC 2+/3+ or *HER2* amplification or elevated circulating serum HER2 extracellular domain when tissue was not available) found that the addition of trastuzumab to cisplatin plus gemcitabine did not significantly improve ORR (41% vs. 36%) [106]. In a single-arm phase II study conducted on patients with *EGFR*-mutant NSCLC showing HER2 IHC ≥ 1 upon progression to EGFR TKI, trastuzumab plus paclitaxel achieved 46% ORR (11 out of 24 patients). Overall response rate was 67% in HER2 IHC 3+ (seven out of 12 patients) [77].

TDM1 achieved an ORR of 20% in HER2 IHC 3+ (4 partial responses out of 20 patients) and no responses in HER2 IHC 2+ (n = 29) [107].

T-DXd was the first ADC to be granted by the FDA and the EMA as agnostic approval for previously treated HER2 overexpressing (IHC 3+) solid tumors [108], based on the results of three phase II trials: DESTINY-PanTumor02 [109], DESTINY-Lung01 [76], DESTINY-CRC02 [110].

DESTINY-PanTumor02 was conducted in previously treated advanced tumors with HER2 IHC 2+/3+. While in the overall population the ORR was 37.1% (n = 99; 95% CI, 31.3–43.2), in the subgroup of patients for which HER2 3+ expression had been confirmed centrally (n = 75), ORR was 61.3% (95% CI, 49.4 to 72.4) [109]. In the DESTINY-Lung01 trial, investigating T-DXd in previously treated, advanced non-squamous NSCLC, one cohort enrolled patients with HER2 IHC 2+ or 3+, with no concomitant *HER2* mutation [76]. In this cohort, ORR was 26.5% (95% CI, 15.0–41.1; 13 of 49 enrolled patients achieved a partial response) in patients treated with the 6.4 mg/kg dose; 34.1% (20.1–50.6; 14 of 41 patients, with two complete responses) in patients treated with the 5.4 mg/kg dose [76]. HER2 IHC 2+ represented 80% of patients (n = 39) of cohort 1, treated with the 6.4 mg/kg dose, and 59% (n = 24) of cohort 1A, treated with the 5.4 mg/kg dose [76]. Fourteen (29%) and seven (17%) patients in cohort 1 and cohort 1A had already received HER2 or EGFR TKIs, respectively. Responses were observed in patients with NSCLC harboring *EGFR* (three out of nine, 33%) or *KRAS* mutations (three out of six, 50%) [76].

The phase Ib DESTINY-Lung03 trial was a multi-arm study investigating T-DXd (4.4 or 5.4 mg/kg) plus durvalumab and cisplatin in Arm 1A, with carboplatin in Arm 1B, or T-DXd 5.4 mg/kg monotherapy in Arm 1D. The study population included patients with advanced or metastatic NSCLC, progressed upon one or two previous treatment lines, with HER2 IHC 2+ or 3+ [111]. Response was evaluated in Arm 1B and 1D: the investigator-assessed ORR was 37.5% (95% CI, 18.8–59.4) with T-DXd plus durvalumab and carboplatin and 44.4% (95% CI, 27.9–61.9) with T-DXd monotherapy [111]. However, based on safety concerns with the combination, the combination will not be further explored.

In the HER2-overexpressing cohort of DESTINY-Lung01, neutropenia remained the most common grade ≥ 3 TRAE, presenting in 12 out of 49 (24%) patients treated with the 6.4 mg/kg dose [76]. Interstitial lung disease or pneumonitis occurred in four (8%) and one (2%) patients treated with T-DXd 6.4 and 5.4 mg/kg, respectively, in the DESTINY-Lung01 trial [76]. One grade 5 febrile neutropenia occurred in a patient treated with T-DXd 4.4 mg/kg plus durvalumab 1120 mg and cisplatin 60 mg/m^2^ in the DESTINY-Lung03 [111]. Drug-related ILD/pneumonitis were reported in three (12.5%, one grade 2 and two grade 5) and two (5.6%, both cases grade 2) patients treated with T-DXd plus durvalumab and carboplatin, and with T-DXd alone, respectively, in the DESTINY-Lung03 study [111].

Two other studies evaluated combinations of T-DXd with ICIs in previously treated NSCLC expressing HER2. In the phase Ib DS8201-A-U106 study, cohort 3 included patients with HER2-expressing NSCLC (1+, 2+, 3+). In this population (n = 22), T-DXd 5.4 mg/kg plus pembrolizumab achieved 12 partial responses (ORR 54.5%, 95%CI, 32.2–75.6) [105]. The phase II HUDSON trial was an umbrella trial enrolling patients who progressed to platinum-based chemotherapy and ICI. Data on 23 patients treated with T-DXd 5.4 mg/kg plus durvalumab reported an ORR of 26.1% (80% CI, 14.3–41.3) and 2.8 (80%CI 2.2–5.5) months of PFS. Grade ≥ 3 pneumonitis occurred in 8% of patients [112].

## 4. Current and Future Challenges in HER2-Mutant and HER2-Overexpressing NSCLC

**Figure 4:**
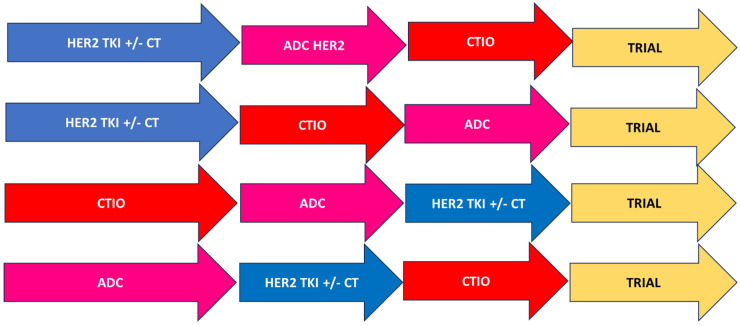
Challenges in the sequential treatment approach in HER2-mutant metastatic NSCLC. (CT: chemotherapy. CTIO: chemoimmunotherapy. TKI: Tyrosine Kinase inhibitor. ADC: antibody–drug conjugates). +/− with / or without.

Based on the ongoing phase III trials, the first challenges will be to elucidate the optimal first-line treatment for each patient and the potential sequential approach (Figure 4). For instance, zongertinib is already approved by the FDA in the first line, and based on oral administration, activity, safety profile, and potential intracranial activity, it seems that HER2 TKIs may become the new SoC in first line worldwide, if available. However, all these TKIs have reported lower activity in non-TKD, suggesting that results of the DESTINY-Lung 04 trial, which include non-TKD HER2 mutations, may help to evaluate the optimal approach for this subgroup of tumors.

It is important to evaluate the potential mechanisms of resistance to explore optimal sequential treatment mechanisms. Resistance or sensitivity to ADCs mainly depends on the sensitivity to their payload [113]; target expression does not necessarily imply sensitivity to the drug, and even when secondary on-target mutations emerge, they might impair trastuzumab binding, while the tumor might retain sensitivity to TKIs [113,114]. Therefore, HER2-TKIs might be used sequentially upon HER2-directed ADCs, as already reported in cohort 5 of the phase I BEAMION-Lung 01 trial [96,97].

It is also important to define the role immunotherapy may play in subsequent lines. Despite having higher TMB than *EGFR*-mutant or *ALK*-rearranged NSCLC, the percentage of cases with TMB-high (≥10 Mut/Mb) was low in real-world series (≈12–25%) [63,64]. One study showed that most patients with TMB-high were male, of older age, and had non-adenocarcinoma tumors with non-exon20ins, therefore differing from the common clinical-molecular profile of the disease [64].

In a real-world cohort from Japan, 53 patients with NSCLC harboring *HER2* mutations received ICI as second line after platinum-doublet failure, with minimal efficacy: PFS was 2.2 months and ORR 0% [64]. Similarly, in a non-Asian cohort study, PFS to ICI was 1.9 months [115]. Combining HER-targeted ADCs with ICIs might be an option, with preliminary clinical activity shown in a phase I trial [105]. Biologically, the tumor microenvironment and immune landscape of *HER2*-altered NSCLC remain poorly characterized, with most available evidence extrapolated from HER2-positive breast and gastroesophageal cancers. Although such cross-histology comparisons have important limitations, *HER2* alterations appear to promote an immunosuppressive microenvironment that may be favorably remodeled through the combination of HER2-directed ADCs and ICIs [116].

Another major challenge, particularly in the context of HER2 overexpression, is that current diagnostic approaches based on IHC provide only a static assessment of a limited tumor region at a single time point [116]. As a result, they fail to adequately capture the spatial and temporal heterogeneity of HER2 expression. This limitation is relevant across different disease contexts. In non–oncogene-addicted tumors, it may hinder the use of HER2 overexpression as a biomarker to guide treatment selection. In oncogene-addicted malignancies, it may also obscure potentially actionable HER2 protein expression, for instance as a target for ADCs after exhaustion of standard targeted therapies. Together with the absence of a standardized HER2 IHC scoring system in NSCLC, these issues represent a major barrier to the reliable use of HER2 overexpression as a predictive biomarker in NSCLC.

Finally, clinical trials are underway to investigate the role of HER2-targeted therapies, including HER2-TKIs, in the adjuvant and perioperative settings (Figure 3). This area is of particular interest because, similarly to other oncogene-driven NSCLCs, *HER2*-mutant tumors may be associated with a substantial risk of central nervous system recurrence, with retrospective data reporting a cumulative incidence of 22% at 5 years [54]. To address this unmet need, the phase III BEAMION-Lung 03 trial (NCT07195695) is evaluating three years of zongertinib versus standard of care treatment, including, when appropriate, immunotherapy, in patients with stage II–IIIB NSCLC harboring *HER2* tyrosine kinase domain mutations following curative-intent surgery, with neoadjuvant chemotherapy and/or immunotherapy or adjuvant chemotherapy.

## 5. Conclusions

*EGFR* and *HER2* exon 20 insertions represent biologically diverse and clinically heterogeneous molecular entities for which effective targeted therapies have only recently become available. Although exon 20 insertion–targeted TKIs generally achieve higher response rates in treatment-naive patients, an optimal sequencing strategy remains to be defined, particularly as these agents appear to retain activity following bispecific antibodies in *EGFR*-mutant disease and HER2-directed ADCs in *HER2*-mutant NSCLC. Ongoing randomized phase III trials comparing targeted therapies alone or in combination with platinum-based chemotherapy, with SOC (chemotherapy with or without immune checkpoint inhibitors), are expected to redefine the first-line treatment landscape. Moreover, whether these malignancies might benefit from targeted agents from earlier stages is still under investigation in clinical trials.

Beyond exon 20 insertions, *HER2* alterations encompass a broader spectrum of molecular abnormalities, such as non-TKD mutations, gene amplification, and protein overexpression. These alterations may occur independently or concurrently, further underscoring the biological complexity of this pathway. In particular, HER2 overexpression is emerging as a histology-agnostic therapeutic target, although its predictive role in NSCLC still requires investigation and standardized biomarker assessment.

## Data Availability

Relevant studies were identified through searches of PubMed and major oncology congresses (including ASCO, ESMO, AACR, ELCC and WCLC), focusing mainly on phase I, II, III clinical trials, translational studies, and key reports on EGFR exon 20 insertion lung cancers as well as HER2 deregulated non–small cell lung cancer.

## References

[1] Oxnard G.R., Lo P.C., Nishino M., Dahlberg S.E., Lindeman N.I., Butaney M., Jackman D.M., Johnson B.E., Jänne P.A. (2013). Natural History and Molecular Characteristics of Lung Cancers Harboring EGFR Exon 20 Insertions. J. Thorac. Oncol..

[2] Riess J.W., Gandara D.R., Frampton G.M., Madison R., Peled N., Bufill J.A., Dy G.K., Ou S.-H.I., Stephens P.J., McPherson J.D. (2018). Diverse EGFR Exon 20 Insertions and Co-Occurring Molecular Alterations Identified by Comprehensive Genomic Profiling of NSCLC. J. Thorac. Oncol..

[3] Cardona A.F., Rojas L., Zatarain-Barrón Z.L., Freitas H.C., Granados S.T., Castillo O., Oblitas G., Corrales L., Castro C.D., Ruiz-Patiño A. (2018). EGFR Exon 20 Insertion in Lung Adenocarcinomas among Hispanics (Geno1.2-CLICaP). Lung Cancer.

[4] Arcila M.E., Nafa K., Chaft J.E., Rekhtman N., Lau C., Reva B.A., Zakowski M.F., Kris M.G., Ladanyi M. (2013). EGFR Exon 20 Insertion Mutations in Lung Adenocarcinomas: Prevalence, Molecular Heterogeneity, and Clinicopathologic Characteristics. Mol. Cancer Ther..

[5] Remon J., Hendriks L.E.L., Cardona A.F., Besse B. (2020). EGFR Exon 20 Insertions in Advanced Non-Small Cell Lung Cancer: A New History Begins. Cancer Treat. Rev..

[6] Yang G., Li J., Xu H., Yang Y., Yang L., Xu F., Xia B., Zhu V.W., Nagasaka M., Yang Y. (2020). EGFR Exon 20 Insertion Mutations in Chinese Advanced Non-Small Cell Lung Cancer Patients: Molecular Heterogeneity and Treatment Outcome from Nationwide Real-World Study. Lung Cancer.

[7] Vyse S., Huang P.H. (2019). Targeting EGFR Exon 20 Insertion Mutations in Non-Small Cell Lung Cancer. Signal Transduct. Target. Ther..

[8] Meador C.B., Sequist L.V., Piotrowska Z. (2021). Targeting EGFR Exon 20 Insertions in Non-Small Cell Lung Cancer: Recent Advances and Clinical Updates. Cancer Discov..

[9] Robichaux J.P., Elamin Y.Y., Tan Z., Carter B.W., Zhang S., Liu S., Li S., Chen T., Poteete A., Estrada-Bernal A. (2018). Mechanisms and Clinical Activity of an EGFR and HER2 Exon 20-Selective Kinase Inhibitor in Non-Small Cell Lung Cancer. Nat. Med..

[10] Yasuda H., Ichihara E., Sakakibara-Konishi J., Zenke Y., Takeuchi S., Morise M., Hotta K., Sato M., Matsumoto S., Tanimoto A. (2021). A Phase I/II Study of Osimertinib in EGFR Exon 20 Insertion Mutation-Positive Non-Small Cell Lung Cancer. Lung Cancer.

[11] Ji J., Aredo J.V., Piper-Vallillo A., Huppert L., Rotow J.K., Husain H., Stewart S., Cobb R., Wakelee H.A., Blakely C.M. (2023). Osimertinib in NSCLC with Atypical EGFR-Activating Mutations: A Retrospective Multicenter Study. JTO Clin. Res. Rep..

[12] van Veggel B., Santos J.F.V.M.R., Hashemi S.M.S., Paats M.S., Monkhorst K., Heideman D.A.M., Groves M., Radonic T., Smit E.F., Schuuring E. (2020). Osimertinib Treatment for Patients with EGFR Exon 20 Mutation Positive Non-Small Cell Lung Cancer. Lung Cancer.

[13] Piotrowska Z., Wang Y., Sequist L.V., Ramalingam S.S. (2020). ECOG-ACRIN 5162: A Phase II Study of Osimertinib 160 Mg in NSCLC with EGFR Exon 20 Insertions. J. Clin. Oncol..

[14] Bazhenova L., Minchom A., Viteri S., Bauml J.M., Ou S.-H.I., Gadgeel S.M., Trigo J.M., Backenroth D., Li T., Londhe A. (2021). Comparative Clinical Outcomes for Patients with Advanced NSCLC Harboring EGFR Exon 20 Insertion Mutations and Common EGFR Mutations. Lung Cancer.

[15] Mountzios G., Planchard D., Metro G., Tsiouda D., Prelaj A., Lampaki S., Shalata W., Riudavets M., Christopoulos P., Girard N. (2023). Molecular Epidemiology and Treatment Patterns of Patients with EGFR Exon 20-Mutant NSCLC in the Precision Oncology Era: The European EXOTIC Registry. JTO Clin. Res. Rep..

[16] Ou S.-H.I., Hong J.-L., Christopoulos P., Lin H.M., Vincent S., Churchill E.N., Soeda J., Kazdal D., Stenzinger A., Thomas M. (2023). Distribution and Detectability of EGFR Exon 20 Insertion Variants in NSCLC. J. Thorac. Oncol..

[17] Viteri S., Minchom A., Bazhenova L., Ou S.-H.I., Bauml J.M., Shell S.A., Schaffer M., Gu J., Rose J.B., Curtin J.C. (2023). Frequency, Underdiagnosis, and Heterogeneity of Epidermal Growth Factor Receptor Exon 20 Insertion Mutations Using Real-World Genomic Datasets. Mol. Oncol..

[18] Park G.-H., Park S., Kim H., Jung H.-A., Sun J.-M., Ahn J.S., Ahn M.-J., Lee S.-H. (2025). Prospective Investigation of Biomarker and Resistance Mechanism Using Longitudinal Cell-Free NGS in Non-Small Cell Lung Cancer with EGFR Exon 20 Insertion Treated with Amivantamab. Eur. J. Cancer.

[19] Elamin Y.Y., Robichaux J.P., Carter B.W., Altan M., Tran H., Gibbons D.L., Heeke S., Fossella F.V., Lam V.K., Le X. (2022). Poziotinib for EGFR Exon 20-Mutant NSCLC: Clinical Efficacy, Resistance Mechanisms, and Impact of Insertion Location on Drug Sensitivity. Cancer Cell.

[20] Le X., Robichaux J.P., Nilsson M., Vijayan R.S.K., Ravichandran A., Wu J., Elamin Y.Y., Hong L., Pei J., He J. (2025). Poziotinib for EGFR Exon 20-Insertion NSCLC: Clinical Efficacy of the Phase 2 ZENITH Trial and Differential Impact of EGFR Exon 20 Insertion Location on Sensitivity. Nat. Commun..

[21] Zhou C., Ramalingam S.S., Kim T.M., Kim S.-W., Yang J.C.-H., Riely G.J., Mekhail T., Nguyen D., Garcia Campelo M.R., Felip E. (2021). Treatment Outcomes and Safety of Mobocertinib in Platinum-Pretreated Patients with EGFR Exon 20 Insertion-Positive Metastatic Non-Small Cell Lung Cancer: A Phase 1/2 Open-Label Nonrandomized Clinical Trial. JAMA Oncol..

[22] Jänne P.A., Wang B.-C., Cho B.C., Zhao J., Li J., Hochmair M., Peters S., Besse B., Pavlakis N., Neal J.W. (2025). First-Line Mobocertinib Versus Platinum-Based Chemotherapy in Patients with EGFR Exon 20 Insertion-Positive Metastatic Non-Small Cell Lung Cancer in the Phase III EXCLAIM-2 Trial. J. Clin. Oncol..

[23] Park K., Haura E.B., Leighl N.B., Mitchell P., Shu C.A., Girard N., Viteri S., Han J.-Y., Kim S.-W., Lee C.K. (2021). Amivantamab in EGFR Exon 20 Insertion-Mutated Non-Small-Cell Lung Cancer Progressing on Platinum Chemotherapy: Initial Results from the CHRYSALIS Phase I Study. J. Clin. Oncol..

[24] Lopez P.G., Girard N., Cho B.C., Sabari J., Spira A., Sanborn R.E., Goto K., Yang J.C.-H., Curtin J., Lyu X. (2023). 30 Long-Term Efficacy, Safety, and Predictors of Response to Amivantamab among Patients with Post-Platinum EGFR Ex20ins-Mutated Advanced NSCLC. J. Thorac. Oncol..

[25] Minchom A., Viteri S., Bazhenova L., Gadgeel S.M., Ou S.-H.I., Trigo J., Bauml J.M., Backenroth D., Bhattacharya A., Li T. (2022). Amivantamab Compared with Real-World Therapies in Patients with Advanced Non-Small Cell Lung Cancer Harboring EGFR Exon 20 Insertion Mutations Who Progressed after Platinum-Based Chemotherapy. Lung Cancer.

[26] Nagasaka M., Goto K., Gomez J., Hida T., Shu C., Lee C.K., Park K., Cho B.C., Lee J., Ou S. (2021). P50.04 Amivantamab in Combination with Chemotherapy in Patients with Advanced Non-Small Cell Lung Cancer (NSCLC). J. Thorac. Oncol..

[27] Zhou C., Tang K.-J., Cho B.C., Liu B., Paz-Ares L., Cheng S., Kitazono S., Thiagarajan M., Goldman J.W., Sabari J.K. (2023). Amivantamab plus Chemotherapy in NSCLC with EGFR Exon 20 Insertions. N. Engl. J. Med..

[28] Zhou C., Agrawal T., Girard N. (2024). VTE with Amivantamab plus Chemotherapy in NSCLC. Reply. N. Engl. J. Med..

[29] Felip E., Shu C.A., Aguilar A., Tang K.-J., Campelo M.R.G., Lee K.-Y., Delmonte A., Sabari J., Girard N., Mansfield A.S. (2024). 2MO Amivantamab plus Chemotherapy vs Chemotherapy as First-Line Treatment in EGFR Exon 20 Insertion-Mutated Advanced NSCLC: Analysis of Post-Progression Endpoints from PAPILLON. ESMO Open.

[30] Spira A.I., Paz-Ares L., Han J.-Y., Shih J.-Y., Mascaux C., Roy U.B., Zugazagoitia J., Kim Y.J., Chiu C.-H., Kim S.-W. (2025). Preventing Infusion-Related Reactions with Intravenous Amivantamab-Results from SKIPPirr, a Phase 2 Study: A Brief Report. J. Thorac. Oncol..

[31] Cho B.C., Li W., Spira A.I., Sauder M., Feldman J., Bozorgmehr F., Mak M., Smith J., Voon P.J., Liu B. (2025). Enhanced Versus Standard Dermatologic Management with Amivantamab-Lazertinib in EGFR-Mutated Advanced NSCLC: The COCOON Global Randomized Controlled Trial. J. Thorac. Oncol..

[32] Lim S.-M., Tan J.-L., Wang J., Paz-Ares L., Yanagitani N., Tang K.-J., Urban D., Scott S.C., Rittmeyer A., Hiret S. (2025). MA08.03 First-Line Subcutaneous Amivantamab Plus Chemotherapy in EGFR Exon 20 Insertion-Mutated Advanced NSCLC: Results from PALOMA-2. J. Thorac. Oncol..

[33] Brazel D., Smith J., Ou S.-H.I., Nagasaka M. (2025). The User’s Guide to Amivantamab. Target. Oncol..

[34] Wang M., Fan Y., Sun M., Wang Y., Zhao Y., Jin B., Hu Y., Han Z., Song X., Liu A. (2024). Sunvozertinib for Patients in China with Platinum-Pretreated Locally Advanced or Metastatic Non-Small-Cell Lung Cancer and EGFR Exon 20 Insertion Mutation (WU-KONG6): Single-Arm, Open-Label, Multicentre, Phase 2 Trial. Lancet Respir. Med..

[35] Yang J.C.-H., Wang M., Doucet L., Fan Y., Lv D., Sun M., Huang D., Greillier L., Planchard D., Hong Q. (2025). Phase II Dose-Randomized Study of Sunvozertinib in Platinum-Pretreated Non-Small Cell Lung Cancer with Epidermal Growth Factor Receptor Exon 20 Insertion Mutations (WU-KONG1B). J. Clin. Oncol..

[36] Yang J.C.-H., Wang M., Chiu C.-H., Hsu P.-C., Mitchell P., Chang C.L., Kim T.M., John T., Greillier L., Bazhenova L. (2023). 1325P Sunvozertinib as First-Line Treatment in NSCLC Patients with EGFR Exon20 Insertion Mutations. Ann. Oncol..

[37] Zhou C., Greillier L., Liu G., John T., Xing L., Kowalski D., Memmott R.M., Yazici O., Sun M., Shu C. (2026). First-Line Sunvozertinib in NSCLC with EGFR Exon 20 Insertion Mutations. N. Engl. J. Med..

[38] Han B., Zhou C., Zheng W., Wu L., Ma Z., Wang H., Yu X., Ding G., Ma D., Nie L. (2023). OA03.04 A Phase 1b Study Of Furmonertinib, an Oral, Brain Penetrant, Selective EGFR Inhibitor, in Patients with Advanced NSCLC with EGFR Exon 20 Insertions. J. Thorac. Oncol..

[39] Cheng Y., Liu Y., Yu Y., Wu L., Luo Y., Li X., Xiao Z., Ren X., Wang X., Lu J. (2025). 1848MO Phase II Study of Firmonertinib in Patients with Previously Treated Advanced/Metastatic Non-Small Cell Lung Cancer (mNSCLC) with EGFR Exon 20 Insertion (Ex20ins) Mutations. Ann. Oncol..

[40] Piotrowska Z., Passaro A., Nguyen D., Ruiter G., Soo R.A., Ho-Fun Lee V., Velcheti V., Tan D.S.-W., Lee S.-H., Kim S.H. (2025). Zipalertinib in Patients with Epidermal Growth Factor Receptor Exon 20 Insertion-Positive Non-Small Cell Lung Cancer Previously Treated with Platinum-Based Chemotherapy with or Without Amivantamab. J. Clin. Oncol..

[41] Piotrowska Z., Nguyen D., Lee V.H.-F., Velcheti V., Passaro A., Lee S.-H., Soo R.A., Wrangle J., Ruiter G., Tan D.S.-W. (2025). MA08.02 Zipalertinib in NSCLC Patients (Pts) with EGFR Exon 20 Insertion (Ex20Ins) Mutations Who Received Prior Amivantamab. J. Thorac. Oncol..

[42] Ohashi K., Yu H.A., Ariyasu R., Zugazagoitia J., Miura S., Ortega Granados A.L.O., Califano R., Spira A.I., Elamin Y.Y., Granier M. (2025). 1847MO Activity of Zipalertinib against Active Central Nervous System (CNS) Metastases in Patients with Non-Small Cell Lung Cancer (NSCLC) Harboring EGFR Exon 20 Insertion (Ex20ins)/Other Uncommon Mutations. Ann. Oncol..

[43] Duan J., Wu L., Yang K., Zhao J., Zhao Y., Dai X., Li M., Xie Y., Yao Y., Zhao M. (2024). Safety, Tolerability, Pharmacokinetics, and Preliminary Efficacy of YK-029A in Treatment-Naive Patients with Advanced NSCLC Harboring EGFR Exon 20 Insertion Mutations: A Phase 1 Trial. J. Thorac. Oncol..

[44] Yang J.-J., Mu Y., Wang Z.-H., Duan J.-C., Zhang Y., Wu L., Zhong H., Zhao J., Yao Y., Wang P. (2026). Andamertinib in Advanced NSCLC with EGFR Exon 20 Insertions After Platinum-Based Chemotherapy or Immunotherapy: Results from the Phase 2 KANNON Study. J. Thorac. Oncol..

[45] Zhang L., Fang W.F., Huang Y., Zhao S., Yu Y., Tang X., Dong X., Zhuang W., Luo F., Wang Q. (2026). 7MO Becotarug (JMT101) and Osimertinib (Osi) in Patients (Pts) with Platinum-Pretreated EGFR Exon 20 Insertion-Mutated (Ex20ins) Non-Small Cell Lung Cancer (NSCLC): Final Overall Survival (OS) and Subgroup Analyses from the BECOME Phase II Study. ESMO Open.

[46] John T., Mohindra N., Hong M.H., Lee K.H., Leighl N.B., Rotow J., Alder L., Dziadziuszko R., Krebs M.G., Sabari J. (2025). LBA13 Enozertinib (ORIC-114), a Highly Selective, Brain Penetrant EGFR and HER2 Inhibitor, in EGFR Exon 20 Mutant NSCLC: Randomized Dose Optimization and CNS Activity. Ann. Oncol..

[47] Zeng L., Song L., Liu L., Wu F., Xu Q., Yan H., Lin S., Jiang W., Wang Z., Deng L. (2024). First-in-Human Phase I Study of BEBT-109 in Previously Treated EGFR Exon 20 Insertion-Mutated Advanced Non-Small Cell Lung Cancer. Med.

[48] Gazzah A., Lin C.-C., Ruiter G., Garrido P., Mazieres J., Felip E., Luo Y.-H., Spigel D.R., Hiret S., Lee K.H. (2025). Trial in Progress: First-in-Human Study of PFL-721/STX-721 in Participants with Locally Advanced or Metastatic Non-Small Cell Lung Cancer Harboring EGFR Exon 20 Insertion Mutations. J. Clin. Oncol..

[49] Robichaux J.P., Le X., Vijayan R.S.K., Hicks J.K., Heeke S., Elamin Y.Y., Lin H.Y., Udagawa H., Skoulidis F., Tran H. (2021). Structure-Based Classification Predicts Drug Response in EGFR-Mutant NSCLC. Nature.

[50] Planchard D., Jänne P.A., Cheng Y., Yang J.C.-H., Yanagitani N., Kim S.-W., Sugawara S., Yu Y., Fan Y., Geater S.L. (2023). Osimertinib with or without Chemotherapy in EGFR-Mutated Advanced NSCLC. N. Engl. J. Med..

[51] Jänne P.A., Planchard D., Kobayashi K., Yang J.C.-H., Liu Y., Valdiviezo N., Kim T.M., Jiang L., Kagamu H., Yanagitani N. (2026). Survival with Osimertinib plus Chemotherapy in EGFR-Mutated Advanced NSCLC. N. Engl. J. Med..

[52] Liao B.-C., Chiang N.-J., Lai C.-L., Liao W.-Y., Wu S.-G., Yang C.-J., Lee M.-H., Chong I.-W., Shen C.-I., Luo Y.-H. (2025). P3.12.27 Resistance Mechanisms of Novel Targeted Therapies to EGFR Exon 20 Insertion Mutation-Positive Non-Small Cell Lung Cancer. J. Thorac. Oncol..

[53] Tsuboi M., Herbst R.S., John T., Kato T., Majem M., Grohé C., Wang J., Goldman J.W., Lu S., Su W.-C. (2023). Overall Survival with Osimertinib in Resected EGFR-Mutated NSCLC. N. Engl. J. Med..

[54] Ghazali N., Feng J., Hueniken K., Khan K., Balaratnam K., Waddell T.K., Yasufuku K., Pierre A., Donahoe L., Wakeam E. (2024). Analysis of Outcomes in Resected Early-Stage NSCLC with Rare Targetable Driver Mutations. Ther. Adv. Med. Oncol..

[55] Fang W., Li X., Wang Q., Meng X., Zheng W., Sun L., Yao W., Zhuang W., Fan Y., Zhuo M. (2025). Sacituzumab Tirumotecan versus Docetaxel for Previously Treated EGFR-Mutated Advanced Non-Small Cell Lung Cancer: Multicentre, Open Label, Randomised Controlled Trial. Br. Med. J..

[56] Fang W., Wu L., Meng X., Yao Y., Zuo W., Yao W., Xie Y., Zhang Y., Cui J., Zhang Y. (2026). Sacituzumab Tirumotecan in EGFR-TKI-Resistant, EGFR-Mutated Advanced NSCLC. N. Engl. J. Med..

[57] Ahn M.-J., Lisberg A., Goto Y., Sands J., Hong M.H., Paz-Ares L., Pons-Tostivint E., Pérol M., Felip E., Sugawara S. (2025). A Pooled Analysis of Datopotamab Deruxtecan in Patients with EGFR-Mutated NSCLC. J. Thorac. Oncol..

[58] Zhang L., Fang W., Cheng Y., Meng X., Yang R., Jiang G., Cui J., He L., Chen P., Zheng W. (2025). Sacituzumab Tirumotecan (Sac-TMT) in Patients (Pts) with Previously Treated Locally Advanced or Metastatic (LA/M) Non-Small Cell Lung Cancer (NSCLC) Harboring Uncommon EGFR Mutations: Preliminary Results from a Phase 2 Study. J. Clin. Oncol..

[59] Zhou H., Zhao H., Hou X., Wang Y., He Z., Li Y., Ma Y., Zhao Y., Huang Y., Chen L. (2026). Izalontamab Brengitecan in Locally Advanced or Metastatic Non-Small Cell Lung Cancer with Actionable Genomic Alterations Outside of Classical EGFR Mutations: A Phase Ib Study. J. Clin. Oncol..

[60] Steuer C.E., Hayashi H., Su W.-C., Nishio M., Johnson M.L., Kim D.-W., Massarelli E., Felip E., Gold K.A., Murakami H. (2025). Patritumab Deruxtecan (HER3-DXd; MK-1022) in Non-Small Cell Lung Cancer After Platinum-Based Chemotherapy and Immunotherapy. J. Clin. Oncol..

[61] Hong L., Patel S., Drusbosky L.M., Xiong Y., Chen R., Geng R., Heeke S., Nilsson M., Wu J., Heymach J.V. (2024). Molecular Landscape of ERBB2 Alterations in 3000 Advanced NSCLC Patients. npj Precis. Oncol..

[62] Lee Y., Lee B., Choi Y.-L., Kang D.-W., Han J. (2024). Clinicopathologic and Molecular Characteristics of HER2 (ERBB2)-Altered Non–Small Cell Lung Cancer: Implications for Precision Medicine. Mod. Pathol..

[63] Lovly C.M., Baik C., Nagasaka M., Patil T., Maruti S.S., Stanhope S., Kaya N.A., Herbertz S., Nordstrom B., Evans K. (2026). Real-World Patient Characteristics, Mutational Landscape, and Outcomes in Advanced/Metastatic HER2-Mutant Non-Small Cell Lung Cancer. JCO Precis. Oncol..

[64] Kato Y., Udagawa H., Matsumoto S., Izumi H., Ohe Y., Kato T., Nishino K., Miyamoto S., Kawana S., Chikamori K. (2024). Efficacy of Immune Checkpoint Inhibitors plus Platinum-Based Chemotherapy as 1st Line Treatment for Patients with Non-Small Cell Lung Cancer Harboring HER2 Mutations: Results from LC-SCRUM-Asia. Lung Cancer.

[65] Reinhorn D., Moskovitz M., Tap W.D., Li B.T. (2025). Targeting HER2 in Lung Cancers: Evolving Treatment Landscape and Drug Development Strategies. Cancer.

[66] Hendriks L.E., Kerr K.M., Menis J., Mok T.S., Nestle U., Passaro A., Peters S., Planchard D., Smit E.F., Solomon B.J. (2023). Oncogene-Addicted Metastatic Non-Small-Cell Lung Cancer: ESMO Clinical Practice Guideline for Diagnosis, Treatment and Follow-Up. Ann. Oncol..

[67] Ren S., Wang J., Ying J., Mitsudomi T., Lee D.H., Wang Z., Chu Q., Mack P.C., Cheng Y., Duan J. (2022). Consensus for HER2 Alterations Testing in Non-Small-Cell Lung Cancer. ESMO Open.

[68] Lee S.H., Jeong H., Kim D.H., Jang S.J., Kim S.-W., Yoon S., Lee D.H. (2024). Comparison of Clinicopathogenomic Features and Treatment Outcomes of EGFR and HER2 Exon 20 Insertion Mutations in Non–Small Cell Lung Cancer: Single-Institution Experience. Cancer Res. Treat..

[69] Rolfo C., Russo A. (2023). Exploiting the Full Potential of Novel Agents Targeting EGFR Exon 20 Insertions in Advanced NSCLC: Next-Generation Sequencing Outperforms Polymerase Chain Reaction–Based Testing. J. Thorac. Oncol..

[70] Li B.T., Ross D.S., Aisner D.L., Chaft J.E., Hsu M., Kako S.L., Kris M.G., Varella-Garcia M., Arcila M.E. (2016). HER2 Amplification and HER2 Mutation Are Distinct Molecular Targets in Lung Cancers. J. Thorac. Oncol..

[71] Kim E.K., Kim K.A., Lee C.Y., Shim H.S. (2017). The Frequency and Clinical Impact of HER2 Alterations in Lung Adenocarcinoma. PLoS ONE.

[72] Leonetti A., Sharma S., Minari R., Perego P., Giovannetti E., Tiseo M. (2019). Resistance Mechanisms to Osimertinib in EGFR-Mutated Non-Small Cell Lung Cancer. Br. J. Cancer.

[73] Shreenivas A., Elliott A., Corbiere T., Chen H.-Z., George B., Kamgar M., Chakrabarti S., Desai A., Bracken-Clarke D., Xue E. (2026). ERBB2/HER2 Landscape and Prognostic Impact: Large-Scale, Real-World Analysis across Solid Cancers. ESMO Open.

[74] Bunn P.A., Helfrich B., Soriano A.F., Franklin W.A., Varella-Garcia M., Hirsch F.R., Baron A., Zeng C., Chan D.C. (2001). Expression of Her-2/Neu in Human Lung Cancer Cell Lines by Immunohistochemistry and Fluorescence in Situ Hybridization and Its Relationship to in Vitro Cytotoxicity by Trastuzumab and Chemotherapeutic Agents. Clin. Cancer Res..

[75] Bartley A.N., Washington M.K., Colasacco C., Ventura C.B., Ismaila N., Benson A.B., Carrato A., Gulley M.L., Jain D., Kakar S. (2017). *HER2* Testing and Clinical Decision Making in Gastroesophageal Adenocarcinoma: Guideline from the College of American Pathologists, American Society for Clinical Pathology, and the American Society of Clinical Oncology. J. Clin. Oncol..

[76] Smit E.F., Felip E., Uprety D., Nagasaka M., Nakagawa K., Paz-Ares Rodríguez L., Pacheco J.M., Li B.T., Planchard D., Baik C. (2024). Trastuzumab Deruxtecan in Patients with Metastatic Non-Small-Cell Lung Cancer (DESTINY-Lung01): Primary Results of the HER2-Overexpressing Cohorts from a Single-Arm, Phase 2 Trial. Lancet Oncol..

[77] De Langen A.J., Jebbink M., Hashemi S.M.S., Kuiper J.L., De Bruin-Visser J., Monkhorst K., Thunnissen E., Smit E.F. (2018). Trastuzumab and Paclitaxel in Patients with EGFR Mutated NSCLC That Express HER2 after Progression on EGFR TKI Treatment. Br. J. Cancer.

[78] Wang Y., Zhang S., Wu F., Zhao J., Li X., Zhao C., Ren S., Zhou C. (2018). Outcomes of Pemetrexed-Based Chemotherapies in HER2-Mutant Lung Cancers. BMC Cancer.

[79] Mazières J., Barlesi F., Filleron T., Besse B., Monnet I., Beau-Faller M., Peters S., Dansin E., Früh M., Pless M. (2016). Lung Cancer Patients with HER2 Mutations Treated with Chemotherapy and HER2-Targeted Drugs: Results from the European EUHER2 Cohort. Ann. Oncol..

[80] Waliany S., Wakelee H., Ramchandran K., Das M., Huang J., Myall N., Li C., Pagtama J., Tisch A.H., Neal J.W. (2022). Characterization of ERBB2 (HER2) Alterations in Metastatic Non-Small Cell Lung Cancer and Comparison of Outcomes of Different Trastuzumab-Based Regimens. Clin. Lung Cancer.

[81] Hainsworth J.D., Meric-Bernstam F., Swanton C., Hurwitz H., Spigel D.R., Sweeney C., Burris H., Bose R., Yoo B., Stein A. (2018). Targeted Therapy for Advanced Solid Tumors on the Basis of Molecular Profiles: Results from MyPathway, an Open-Label, Phase IIa Multiple Basket Study. J. Clin. Oncol..

[82] Hirsh V. (2015). Next-Generation Covalent Irreversible Kinase Inhibitors in NSCLC: Focus on Afatinib. BioDrugs.

[83] Dziadziuszko R., Smit E.F., Dafni U., Wolf J., Wasąg B., Biernat W., Finn S.P., Kammler R., Tsourti Z., Rabaglio M. (2019). Afatinib in NSCLC with HER2 Mutations: Results of the Prospective, Open-Label Phase II NICHE Trial of European Thoracic Oncology Platform (ETOP). J. Thorac. Oncol..

[84] Li B.T., Gandhi L., Ravichandran V., Besse B., Mazières J., Shapiro G.I., Boni V., Waqar S.N., Park H., Quinn D.I. (2026). Efficacy of Neratinib-Based Therapy in ERBB2-Mutant Lung Adenocarcinomas: Findings from 2 International Phase 2 Studies. Clin. Lung Cancer.

[85] Kris M.G., Camidge D.R., Giaccone G., Hida T., Li B.T., O’Connell J., Taylor I., Zhang H., Arcila M.E., Goldberg Z. (2015). Targeting HER2 Aberrations as Actionable Drivers in Lung Cancers: Phase II Trial of the Pan-HER Tyrosine Kinase Inhibitor Dacomitinib in Patients with HER2-Mutant or Amplified Tumors. Ann. Oncol..

[86] Elamin Y.Y., Robichaux J.P., Carter B.W., Altan M., Gibbons D.L., Fossella F.V., Lam V.K., Patel A.B., Negrao M.V., Le X. (2022). Poziotinib for Patients with HER2 Exon 20 Mutant Non-Small-Cell Lung Cancer: Results from a Phase II Trial. J. Clin. Oncol..

[87] Le X., Cornelissen R., Garassino M., Clarke J.M., Tchekmedyian N., Goldman J.W., Leu S.-Y., Bhat G., Lebel F., Heymach J.V. (2022). Poziotinib in Non-Small-Cell Lung Cancer Harboring HER2 Exon 20 Insertion Mutations After Prior Therapies: ZENITH20-2 Trial. J. Clin. Oncol..

[88] Cornelissen R., Prelaj A., Sun S., Baik C., Wollner M., Haura E.B., Mamdani H., Riess J.W., Cappuzzo F., Garassino M.C. (2023). Poziotinib in Treatment-Naive NSCLC Harboring HER2 Exon 20 Mutations: ZENITH20-4, A Multicenter, Multicohort, Open-Label, Phase 2 Trial (Cohort 4). J. Thorac. Oncol..

[89] Zhou C., Li X., Wang Q., Gao G., Zhang Y., Chen J., Shu Y., Hu Y., Fan Y., Fang J. (2020). Pyrotinib in HER2-Mutant Advanced Lung Adenocarcinoma After Platinum-Based Chemotherapy: A Multicenter, Open-Label, Single-Arm, Phase II Study. J. Clin. Oncol..

[90] Liu S.-Y.M., Tu H.-Y., Wei X.-W., Yan H.-H., Dong X.-R., Cui J.-W., Zhou Z., Xu C.-R., Zheng M.-Y., Li Y.-S. (2023). First-Line Pyrotinib in Advanced HER2-Mutant Non-Small-Cell Lung Cancer: A Patient-Centric Phase 2 Trial. Nat. Med..

[91] Han H., Li S., Chen T., Fitzgerald M., Liu S., Peng C., Tang K.H., Cao S., Chouitar J., Wu J. (2021). Targeting HER2 Exon 20 Insertion-Mutant Lung Adenocarcinoma with a Novel Tyrosine Kinase Inhibitor Mobocertinib. Cancer Res..

[92] Kanemura H., Tanizaki J., Matsumoto K., Masuishi T., Isobe T., Kenmotsu H., Sunakawa Y., Shimizu T., Chiba Y., Yamamoto N. (2026). Phase Ia/Ib Trial of the Safety and Efficacy of Mobocertinib in Combination with T-DM1 for Patients with HER2-Mutant Solid Tumors (WJOG16022M). Eur. J. Cancer.

[93] Siegel F., Siegel S., Kotýnková K., Karsli Uzunbas G., Korr D., Tomono H., Andersen S., Denney D., Berger M., Schulze V.K. (2026). Sevabertinib, a Reversible HER2 Inhibitor with Activity in Lung Cancer. Cancer Discov..

[94] Le X., Kim T.M., Loong H.H., Prelaj A., Goh B.C., Li L., Fang Y., Lu S., Dong X., Wu L. (2025). Sevabertinib in Advanced HER2-Mutant Non-Small-Cell Lung Cancer. N. Engl. J. Med..

[95] Le X., Goto K., Lu S., Brase J.C., Xu J., Pu S.-F., Przybytniak I., Mongay Soler L., Garon E.B., Passaro A. (2025). SOHO-02: Phase III Trial of BAY 2927088 in Patients with Locally Advanced or Metastatic NSCLC with HER2-Activating Mutations. J. Clin. Oncol..

[96] Heymach J.V., Ruiter G., Ahn M.-J., Girard N., Smit E.F., Planchard D., Wu Y.-L., Cho B.C., Yamamoto N., Sabari J.K. (2025). Zongertinib in Previously Treated HER2-Mutant Non-Small-Cell Lung Cancer. N. Engl. J. Med..

[97] Heymach J.V., Ruiter G., Ahn M.-J., Girard N., Smit E., Planchard D., Wu Y.-L., Cho B.C., Yamamoto N., Sabari J.K. (2025). Abstract CT050: Zongertinib in Patients with Pretreated HER2-Mutant Advanced NSCLC: Beamion LUNG-1. Cancer Res..

[98] Heymach J.V., Yamamoto N., Girard N., Ruiter G., Smit E.F., Planchard D., Nadal E., Wu Y.-L., Zugazagoitia J., Tu H.-Y. (2026). First-Line Zongertinib in Advanced HER2-Mutant Non-Small-Cell Lung Cancer. N. Engl. J. Med..

[99] Li B.T., Shen R., Buonocore D., Olah Z.T., Ni A., Ginsberg M.S., Ulaner G.A., Offin M., Feldman D., Hembrough T. (2018). Ado-Trastuzumab Emtansine for Patients with HER2-Mutant Lung Cancers: Results from a Phase II Basket Trial. J. Clin. Oncol..

[100] Li B.T., Smit E.F., Goto Y., Nakagawa K., Udagawa H., Mazières J., Nagasaka M., Bazhenova L., Saltos A.N., Felip E. (2022). Trastuzumab Deruxtecan in HER2-Mutant Non-Small-Cell Lung Cancer. N. Engl. J. Med..

[101] Goto K., Goto Y., Kubo T., Ninomiya K., Kim S.-W., Planchard D., Ahn M.-J., Smit E.F., de Langen A.J., Pérol M. (2023). Trastuzumab Deruxtecan in Patients with *HER2*-Mutant Metastatic Non–Small-Cell Lung Cancer: Primary Results from the Randomized, Phase II DESTINY-Lung02 Trial. J. Clin. Oncol..

[102] Jänne P.A., Planchard D., Goto K., Smit E.F., de Langen A.J., Goto Y., Ninomiya K., Kubo T., Pérol M., Felip E. (2025). Trastuzumab Deruxtecan for ERBB2-Mutant Metastatic Non-Small Cell Lung Cancer with or Without Brain Metastases: A Secondary Analysis of Randomized Clinical Trials. JAMA Netw. Open.

[103] Li Z., Wang Y., Sun Y., Wang L., Li X., Sun L., He Z., Yang H., Wang Y., Wang Q. (2025). Trastuzumab Rezetecan, a HER2-Directed Antibody-Drug Conjugate, in Patients with Advanced HER2-Mutant Non-Small-Cell Lung Cancer (HORIZON-Lung): Phase 2 Results from a Multicentre, Single-Arm Study. Lancet Oncol..

[104] Zhang L., Fan Y., Zhao Y., Huang Z., Guo Q., Li J., Huang S., Li D., Liu X., Shi H. (2025). 160MO BL-M07D1, a Novel Anti-HER2 Antibody-Drug Conjugate (ADC), in Subjects with Metastatic HER2-Mutant Non-Small Cell Lung Cancer (NSCLC). Ann. Oncol..

[105] Italiano A., Besse B., Borghaei H., Popat S., Palacios G.A., Goncalves A., Meurer M., Mazieres J., Chouaid C., García J.S. (2024). 118MO Trastuzumab Deruxtecan (T-DXd) and Pembrolizumab in Immuno-Oncology (IO)-Naive HER2-Expressing or HER2-Mutant Non-Small Cell Lung Cancer (NSCLC): Interim Analysis of a Phase Ib Study. Immuno-Oncol. Technol..

[106] Gatzemeier U., Groth G., Butts C., Van Zandwijk N., Shepherd F., Ardizzoni A., Barton C., Ghahramani P., Hirsh V. (2004). Randomized Phase II Trial of Gemcitabine–Cisplatin with or without Trastuzumab in HER2-Positive Non-Small-Cell Lung Cancer. Ann. Oncol..

[107] Peters S., Stahel R., Bubendorf L., Bonomi P., Villegas A., Kowalski D.M., Baik C.S., Isla D., Carpeno J.D.C., Garrido P. (2019). Trastuzumab Emtansine (T-DM1) in Patients with Previously Treated HER2-Overexpressing Metastatic Non–Small Cell Lung Cancer: Efficacy, Safety, and Biomarkers. Clin. Cancer Res..

[108] Westphalen C.B., Martins-Branco D., Beal J.R., Cardone C., Coleman N., Schram A.M., Halabi S., Michiels S., Yap C., André F. (2024). The ESMO Tumour-Agnostic Classifier and Screener (ETAC-S): A Tool for Assessing Tumour-Agnostic Potential of Molecularly Guided Therapies and for Steering Drug Development. Ann. Oncol..

[109] Meric-Bernstam F., Makker V., Oaknin A., Oh D.-Y., Banerjee S., González-Martín A., Jung K.H., Ługowska I., Manso L., Manzano A. (2024). Efficacy and Safety of Trastuzumab Deruxtecan in Patients with HER2-Expressing Solid Tumors: Primary Results from the DESTINY-PanTumor02 Phase II Trial. J. Clin. Oncol..

[110] Raghav K., Siena S., Takashima A., Kato T., Van den Eynde M., Pietrantonio F., Komatsu Y., Kawakami H., Peeters M., Andre T. (2024). Trastuzumab Deruxtecan in Patients with HER2-Positive Advanced Colorectal Cancer (DESTINY-CRC02): Primary Results from a Multicentre, Randomised, Phase 2 Trial. Lancet Oncol..

[111] Planchard D., Kim H.R., Suksombooncharoen T., Li R., Cortinovis D., Han J.-Y., Samol J., Runglodvatana Y., Lee K.-Y., Chang G.-C. (2026). Trastuzumab Deruxtecan in Patients with HER2-Overexpressing NSCLC: Results from Part 1 of the Open-Label, Multicenter, Phase 1b DESTINY-Lung03 Trial. J. Thorac. Oncol..

[112] Cheema P., Hartl S., Koczywas M., Hochmair M., Shepherd F.A., Chu Q., Galletti G., Gustavson M., Iyer S., Carl Barrett J. (2023). 695 Efficacy and Safety of Trastuzumab Deruxtecan (T-DXd) with Durvalumab in Patients with Non-Small Cell Lung Cancer (HER2 Altered NSCLC) Who Progressed on Anti-PD1/PD-L1 Therapy (HUDSON). Proceedings of the Regular and Young Investigator Award Abstracts.

[113] Zullo L., Bortolot M., Ogliari F.R., Saw S.P.L., van Geel R., Ter Heine R., Sadowska A., Remon J., Hendriks L. (2026). Antibody-Drug Conjugates in Non-Small Cell Lung Cancer. Drugs.

[114] Nilsson M.B., Le X., Poteete A., Yu X., He J., Huang Q., Shibata Y., Liu X., Moran C., Alizadeh A.A. (2026). Loss of Payload Sensitivity and Other Mechanisms of Resistance to T-DXd in HER2-Mutant NSCLC: Implications for Subsequent Responsiveness to HER2 TKIs. J. Thorac. Oncol..

[115] Negrao M.V., Skoulidis F., Montesion M., Schulze K., Bara I., Shen V., Xu H., Hu S., Sui D., Elamin Y.Y. (2021). Oncogene-Specific Differences in Tumor Mutational Burden, PD-L1 Expression, and Outcomes from Immunotherapy in Non-Small Cell Lung Cancer. J. Immunother. Cancer.

[116] Tillman L., Pusztai L., Wander S.A., Sundar R., Klempner S.J. (2026). Reimagining HER2 Therapy: Bridging Oncogene Addiction and Immune Modulation. Cancer Cell.

